# Investigation of the Performance and Mechanism of an *N*-Doped Monolithic Fe/Ni-Based Catalyst for PMS Activation Toward Chlortetracycline Degradation in Water

**DOI:** 10.3390/molecules31162884

**Published:** 2026-08-18

**Authors:** Yiqiong Yang, Juan Han, Cui Wang, Pingchuan Yang, Panchen Li, Xiaodong Zhang

**Affiliations:** School of Environment and Architecture, University of Shanghai for Science and Technology, Shanghai 200093, China; yangyiqiong@usst.edu.cn (Y.Y.); 18298045414@163.com (J.H.); wangcui031129@163.com (C.W.); 2435052526@st.usst.edu.cn (P.Y.); lianger@126.com (P.L.)

**Keywords:** metal–organic frameworks, peroxymonosulfate, chlortetracycline, nickel–iron foam

## Abstract

MOF-derived catalysts have considerable potential for aqueous contaminant control, but their practical application is often constrained by the aggregation and difficult recovery of powder catalysts. In this study, a self-supporting *N*-doped Fe/Ni-based monolithic catalyst, denoted N-101-NFF, was fabricated through the in situ growth of an Fe-based MOF precursor on nickel–iron foam followed by pyrolysis. Under the conditions of 50 mg/L chlortetracycline (CTC), 0.08 mmol/L peroxymonosulfate (PMS), and an effective catalyst area of 1 cm^2^, N-101-NFF degraded 90.3% of CTC within 60 min and maintained a degradation efficiency of 88.6% after five consecutive cycles. Quenching experiments and EPR analysis indicated the involvement of •OH, SO_4_•^−^, O_2_•^−^, and ^1^O_2_ in CTC degradation. Electrochemical measurements indicated improved interfacial charge-transfer characteristics, while post-reaction XPS analysis revealed changes in the Fe and Ni valence states and surface N- and O-containing groups, supporting the involvement of Fe and Ni redox cycling and these surface functionalities in PMS activation. In a fixed-bed reactor, the system maintained more than 86% CTC removal over 24 h of continuous-flow operation at a CTC feed rate of 200 mL/h. These results highlight the potential of N-101-NFF as a recoverable monolithic catalyst for PMS-based treatment of antibiotic-contaminated water.

## 1. Introduction

Antibiotic residues have become an important class of emerging aquatic pollutants because they are frequently detected, poorly eliminated in conventional treatment processes, and able to exert long-term selective pressure in receiving environments [1]. Continuous discharge of antibiotic-containing wastewater may enrich residual antibiotics, resistant bacterial populations and associated resistance genes, thereby increasing ecological risks and potential human health risks. Tetracycline antibiotics (TCs) are of particular concern owing to their broad antibacterial spectrum and extensive consumption in medical, livestock, and aquaculture applications [2]. Chlortetracycline (CTC), one representative TC compound, is widely used in animal production and can enter water bodies through wastewater discharge and agricultural runoff. Therefore, developing efficient treatment systems for CTC-contaminated water is necessary for antibiotic pollution control. Advanced oxidation processes driven by sulfate radicals have received growing research interest as effective strategies for antibiotic degradation from aqueous environments. Compared with hydroxyl radicals, sulfate radicals generally possess a relatively long lifetime, broad pH tolerance, and high oxidative capability [3]. Peroxymonosulfate (PMS) is a common precursor for generating reactive oxygen species and can be activated by heat, light, ultrasound, electrochemical input, or catalysts. Among these activation routes, heterogeneous catalysis is more suitable for engineering application because it can operate under mild conditions and allows catalyst separation or reuse.

Metal–organic frameworks (MOFs) are crystalline porous coordination networks assembled from metal nodes and organic ligands, whose well-defined architectures offer considerable flexibility for tailoring catalytic active sites [4]. With the rapid progress of MOFs chemistry, pore environments, metal centers, and functional groups can be deliberately regulated, providing versatile platforms for environmental catalysis. These features, together with high surface area and tunable composition, make MOFs attractive precursors or platforms for environmental catalysis. In particular, MOF-derived materials can preserve part of the structural advantages of the parent framework while generating conductive carbon matrices and dispersed metal species after thermal conversion. Such characteristics are beneficial for pollutant adsorption, electron transfer, and oxidant activation [5]. Nitrogen incorporation is another effective way to tailor the electronic structure of carbon-based MOFs derivatives. N-containing sites can redistribute local charge density and promote interfacial electron transfer, thereby improving PMS activation efficiency [6]. However, most MOF-derived catalysts are still prepared as powders, which are prone to aggregation and difficult to recover from treated water. Constructing MOF-derived catalysts directly on macroscopic conductive substrates is therefore a practical approach for improving recyclability and moving these materials closer to continuous-flow operation.

In this work, a self-supporting *N*-doped Fe/Ni catalyst (N-101-NFF) was prepared through the in situ anchoring of an Fe-based MOF precursor onto nickel–iron foam, followed by pyrolysis. Unlike conventional powder catalysts, the monolithic architecture integrates recoverability, accessible catalytic interfaces, and improved mass transfer within a macroscopic framework, which is beneficial for practical PMS-based oxidation processes. The physicochemical properties of N-101-NFF were systematically characterized by XRD, SEM, TEM, FT-IR, and XPS. Its catalytic performance toward CTC degradation was evaluated under different PMS dosages, pyrolysis temperatures, initial pH values, inorganic anions, humic acid concentrations, and water matrices. Scavenging experiments, EPR analysis, electrochemical measurements, and post-reaction XPS were further combined to clarify the PMS activation mechanism. In addition, the continuous-flow applicability of N-101-NFF was examined in a fixed-bed system, providing further evidence for the practical potential of MOF-derived monolithic catalysts in antibiotic-contaminated water treatment.

## 2. Results and Discussion

### 2.1. Physicochemical Characterizations

**Figure 1:**
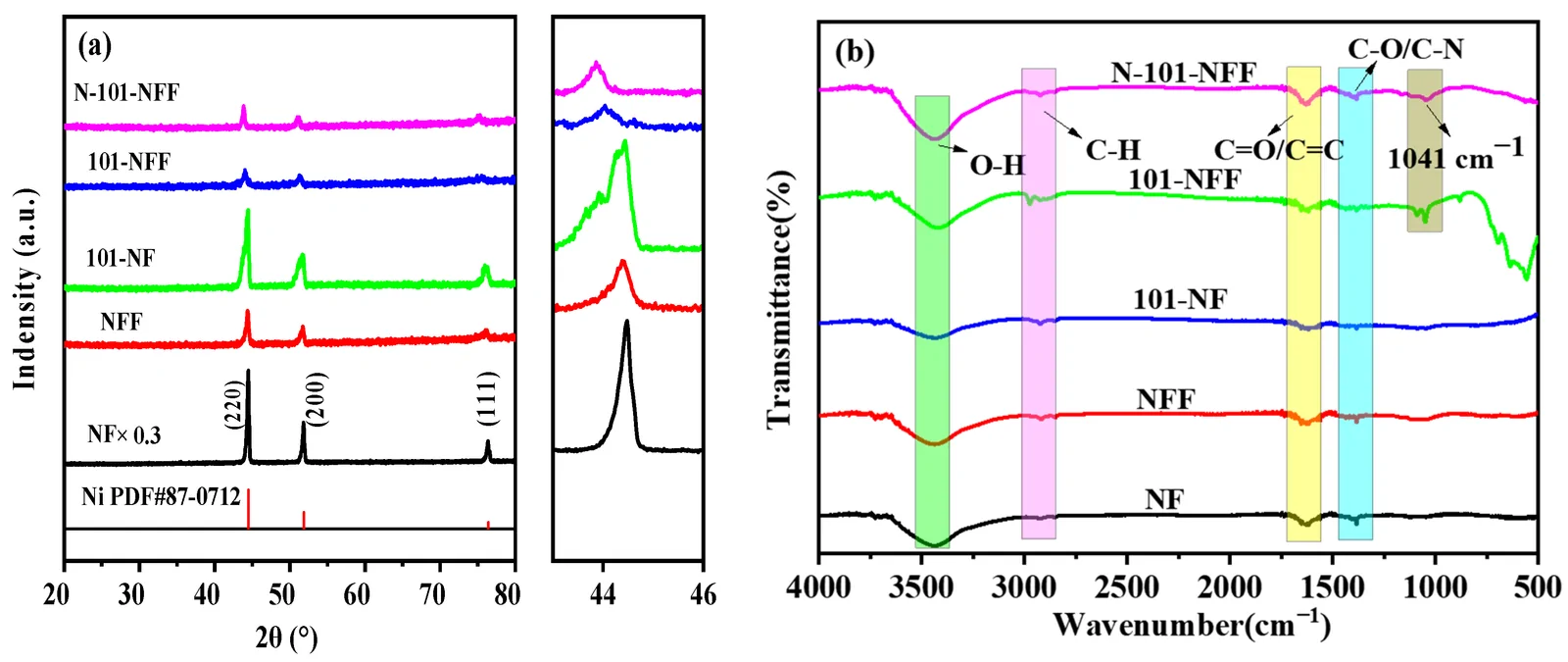
X-ray diffraction (XRD) patterns and the corresponding enlarged view over 2θ = 43–46° (**a**), and (**b**) Fourier-transform infrared (FT-IR) spectra of nickel foam (NF), nickel–iron foam (NFF), the MIL-101(Fe)-derived catalyst supported on NF (101-NF), the MIL-101(Fe)-derived catalyst supported on NFF (101-NFF), and the *N*-doped MIL-101(Fe)-derived catalyst supported on NFF (N-101-NFF).

The XRD patterns of NF, NFF, 101-NF, 101-NFF, and N-101-NFF are shown in Figure 1a. All samples displayed three dominant diffraction peaks at approximately 44.3°, 51.7°, and 76.2°, which can be attributed to the (111), (200), and (220) crystal plane reflections of the face-centered cubic nickel phase (Ni PDF#87-0712). These dominant reflections indicate that the metallic foam substrate remained the principal crystalline phase detected by XRD. No distinct reflections attributable to separate Fe-containing crystalline phases were resolved. This may be related to the confinement of the Fe-containing species within a relatively thin MOF-derived surface layer, together with their low loading, high dispersion, or limited long-range order, which could render the corresponding diffraction signals too weak to be resolved against the intense substrate reflections. Therefore, the XRD results alone do not confirm the formation of a separate Fe-containing crystalline phase. After MOF-derived modification, the relative intensities of the dominant Ni reflections decreased, which may reflect changes in the contribution of the metallic substrate to the overall diffraction pattern after surface-layer formation. A slight shift to lower 2θ values was also observed after Fe-containing modification, possibly reflecting a subtle change in the lattice environment of the Ni-based phase [7].

The formation of an Fe-containing MOF-derived surface layer was further supported by complementary characterization results. SEM and TEM revealed polyhedral particles anchored to the foam framework, EDS elemental mapping confirmed the coexistence of Fe, Ni, C, N, and O, and XPS supported the presence of Fe-containing surface species and Fe-N coordination environments. Taken together, these results support the presence of Fe-containing species within the MOF-derived surface layer on the metallic substrate, although no separate Fe-containing crystalline phase was resolved by XRD.

The FT-IR spectra in Figure 1b provide information on the surface functional groups and bonding environments of the samples. A broad absorption band centered at approximately 3487 cm^−1^ is ascribed to O-H stretching vibrations associated with surface hydroxyl groups and physically adsorbed water. The bands at 1632 and 1376 cm^−1^ are tentatively associated with residual oxygen-containing groups and carbonaceous structures. Given the pyrolysis of the MOF precursor at 700 °C, substantial decomposition and carbonization of the organic linker are expected, and these bands are therefore more reasonably interpreted as arising from the resulting carbonaceous surface rather than intact linker-derived carboxylate species [8,9]. In addition, the absorption feature near 1041 cm^−1^ may contain overlapping contributions from oxygen-containing and metal-oxygen-related surface vibrations and is therefore not assigned to Fe-OH alone [10]. Compared with NF and NFF, the spectral variations in this spectral region for 101-NFF and N-101-NFF may reflect differences in the relative contributions of these surface vibrations. Similar oxygen-containing surface functionalities have also been reported in NH_2_-MIL-101(Fe/Ce)-based composites [11,12]. The absence of an obvious feature near 1041 cm^−1^ for 101-NF may be related to the weak intensity of the corresponding vibrations and/or overlap with adjacent bands. Overall, the FT-IR results indicate the presence of surface hydroxyl and oxygen-containing functionalities. Together with the surface morphology observed by SEM/TEM, the elemental distribution revealed by EDS mapping, and the surface chemical states identified by XPS, these results are consistent with the formation of a MOF-derived catalytic surface layer on the foam substrate.

**Figure 2:**
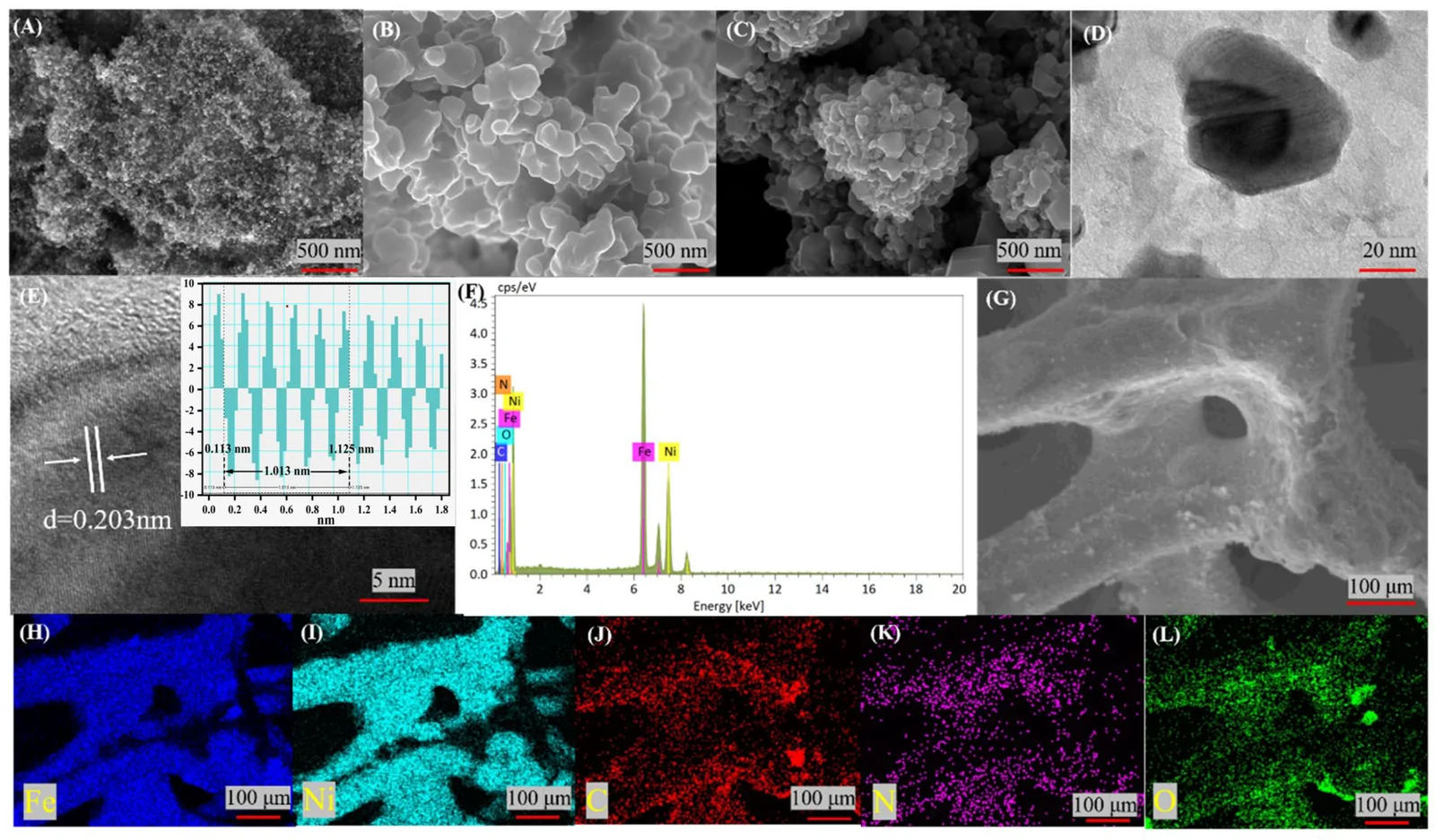
Scanning electron microscopy (SEM) images of 101-NF (**A**), 101-NFF (**B**), and N-101-NFF (**C**); transmission electron microscopy (TEM) image (**D**), high-resolution TEM (HRTEM) image (**E**), energy-dispersive X-ray spectroscopy (EDS) spectrum (**F**), and elemental mapping images (**G**–**L**) of N-101-NFF.

The morphology of the as-prepared catalysts was examined by SEM and TEM, as shown in Figure 2A–L. From the SEM micrographs in Figure 2A–C, distinct surface morphologies were observed for the prepared samples. Compared with 101-NF, which exhibited a rough surface decorated with small particles, 101-NFF and N-101-NFF displayed larger polyhedral particles uniformly anchored on the foam framework, consistent with previously reported Fe-based MOF-derived morphologies [13,14]. These observations indicate that nickel–iron foam provides more favorable nucleation and growth sites for MOF-derived species than nickel foam. Therefore, NFF was selected as the supporting framework for constructing the *N*-doped monolithic catalyst, and the resulting N-101-NFF was subjected to further characterization and catalytic evaluation.

As shown in the HRTEM image of N-101-NFF (Figure 2E), distinct lattice fringes with a spacing of 0.203 nm were observed, which are reasonably indexed to the (111) plane of the Ni-Fe alloy, in agreement with the XRD analysis [15]. In addition, lattice fringes with an interplanar spacing of 0.36 nm can be observed at the nanoparticle edges, which can be assigned to the enlarged (002) interlayer spacing of graphitic carbon. The interlayer spacing of 0.36 nm exceeds that of ideal graphite (ca. 0.34 nm), which may be associated with lattice distortion, structural defects, or expanded stacking of graphitic carbon layers [16]. Compared to ideal graphitic carbon with a corresponding distance of 0.34 nm [17], the current graphitic carbon exhibits a larger layer spacing, which has also been observed in other reported carbon materials [18,19]. EDS and elemental mapping (Figure 2F–L) confirmed the coexistence of Fe, Ni, C, N, and O on N-101-NFF, matching the designed elemental composition. Taken together, the anchored surface particles observed by SEM/TEM, the coexistence of Fe, Ni, C, N, and O revealed by EDS elemental mapping, and the corresponding surface chemical states identified by XPS support the formation of the MOF-derived catalytic surface layer on the foam substrate.

**Figure 3:**
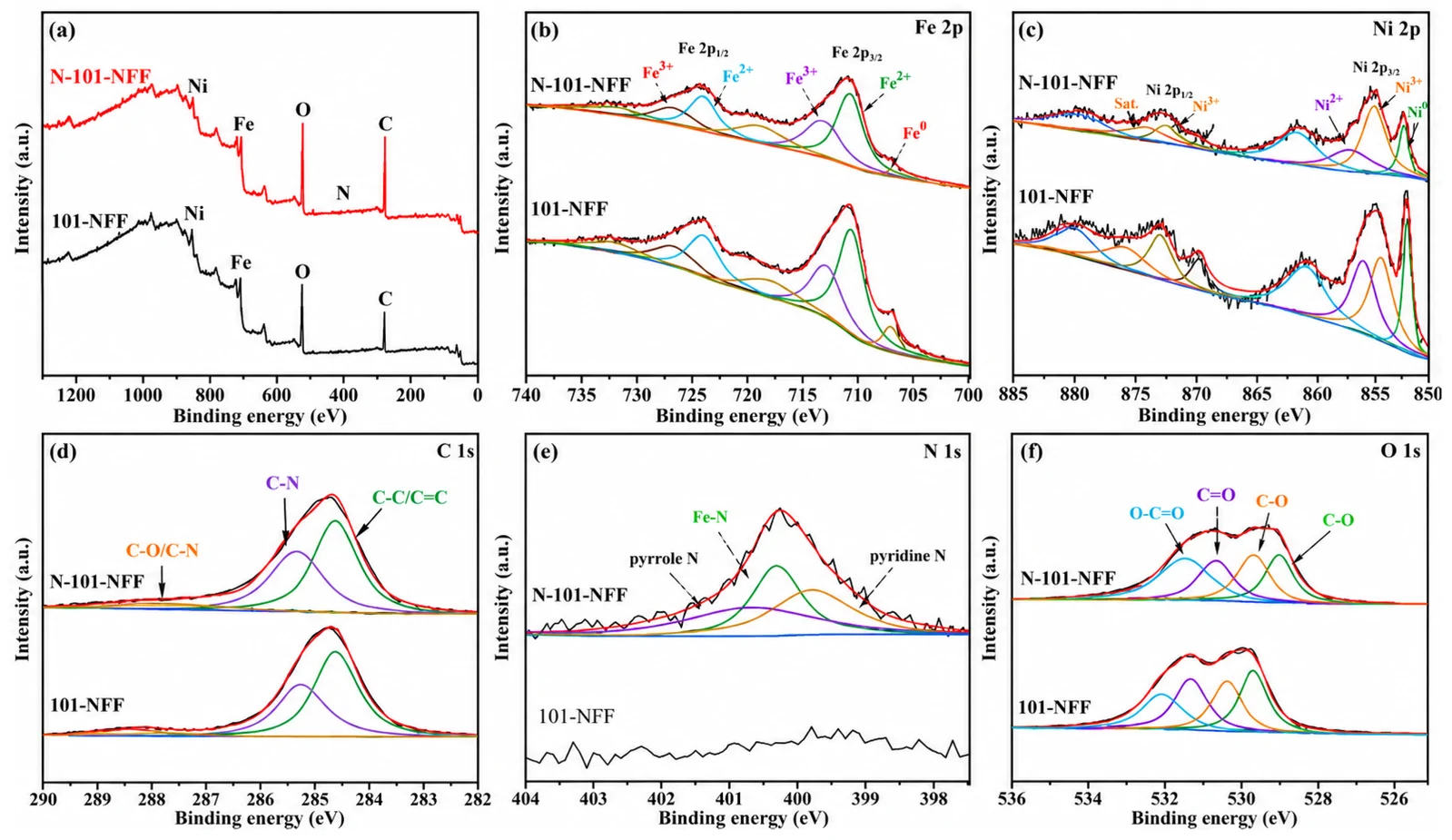
X-ray photoelectron spectroscopy (XPS) survey spectra (**a**) and high-resolution Fe 2p (**b**), Ni 2p (**c**), C 1s (**d**), N 1s (**e**), and O 1s (**f**) spectra of 101-NFF and N-101-NFF.

XPS measurements were employed to clarify the surface chemistry of N-101-NFF. The full-scan XPS spectrum in Figure 3a exhibits distinct Ni, Fe, O, N, and C signals for N-101-NFF, supporting the expected surface elemental composition of the catalyst. In the high-resolution Fe 2p spectrum (Figure 3b), the peaks centered at 711.8 and 723.6 eV corresponded to Fe^2+^, while the signals around 713 and 727 eV were assigned to Fe^3+^ species, suggesting the presence of mixed-valence iron species on the N-101-NFF surface. The Ni 2p spectrum (Figure 3c) contained metallic Ni signals at 852.5 and 870.6 eV and could be deconvoluted into Ni^0^, Ni^2+^, and Ni^3+^ species. For C 1s (Figure 3d), the peaks at 284.5, 285.4, and 287.7 eV corresponded to C=C/C-C, C=N, and C-O/C-N groups, respectively. In addition, as shown in the N 1s spectrum in Figure 3e, N-101-NFF exhibited a distinct N 1s signal, whereas 101-NFF showed no obvious N 1s peak, indicating the successful introduction of N species. The N 1s spectrum of N-101-NFF was resolved into three characteristic contributions centered at 398.8, 399.7, and 400.6 eV, corresponding to pyridinic N, Fe-N coordination, and pyrrolic N, respectively. The O 1s profile in Figure 3f showed four oxygen-containing species, including C=O, C-O, C-C=O, and O-C=O, with binding energies of 528.7, 529.6, 530.6, and 531.4 eV, respectively. Together with the Fe and Ni spectra, these findings indicate that N-containing coordination sites and oxygenated surface groups were successfully introduced into the Fe/Ni-based catalyst.

### 2.2. Catalytic Performance

**Figure 4:**
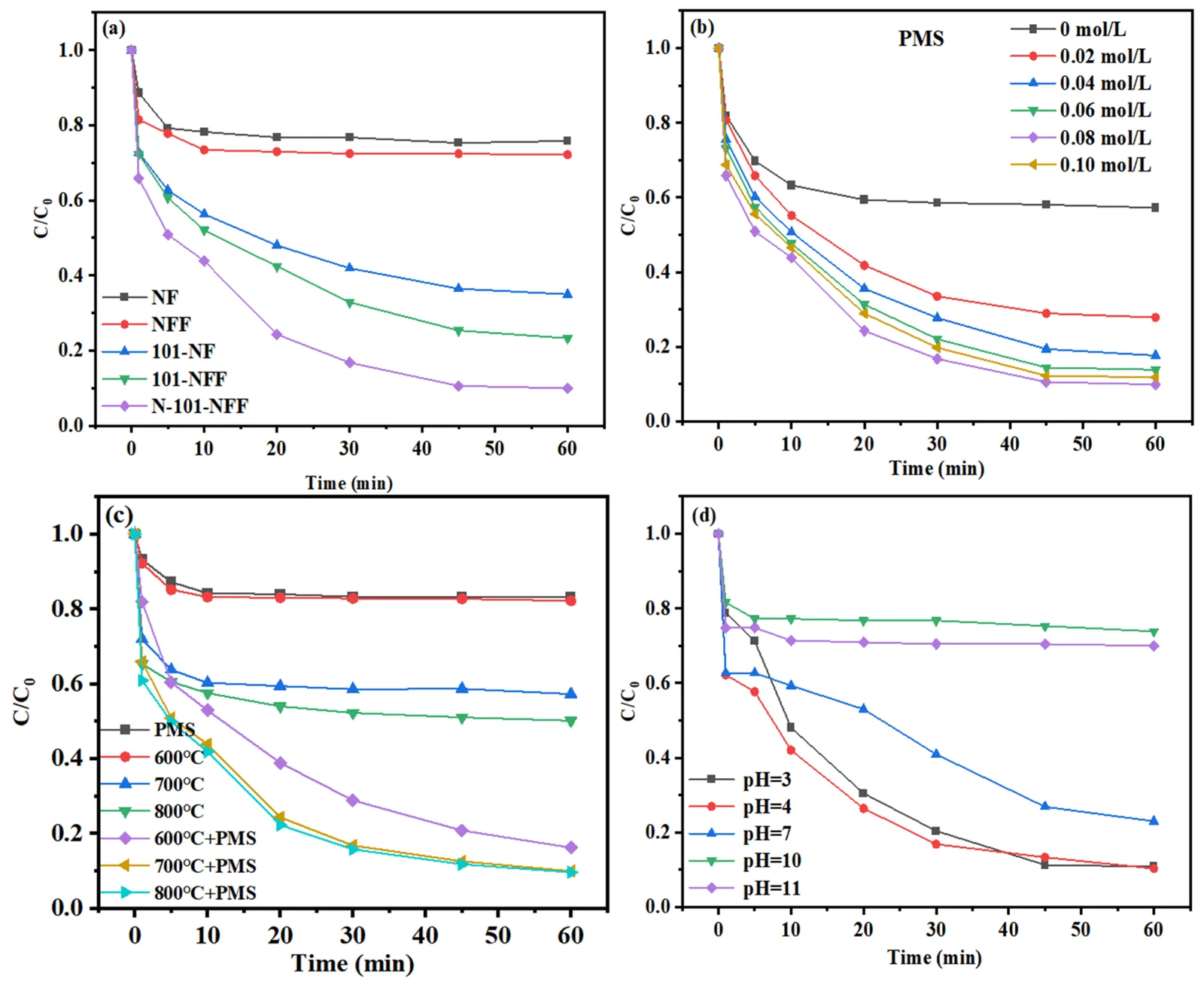
Effects of catalyst type (**a**), PMS dosage (**b**), calcination temperature (**c**), and initial pH (**d**) on CTC degradation in the N-101-NFF/PMS system. (CTC = 50 mg/L, PMS = 0.08 mmol/L, and N-101-NFF = 1 cm^2^ unless otherwise specified).

As shown in Figure 4a, pristine NF and NFF exhibited limited PMS activation ability, leading to poor CTC removal. After MOF-derived modification, both 101-NF and 101-NFF showed enhanced catalytic activity, suggesting that the derived surface layer contributed to PMS activation. Notably, N-101-NFF achieved the highest CTC removal efficiency among all tested catalysts, indicating that N-containing sites and the nickel–iron foam framework jointly promoted the catalytic process. Therefore, N-101-NFF was selected for the subsequent parameter optimization and mechanistic investigations.

### 2.3. Reaction Parameters—Regulated CTC Removal

#### 2.3.1. Effect of PMS Dosage

The effect of PMS dosage on CTC degradation in the N-101-NFF/PMS system is shown in Figure 4b. In the absence of PMS, only limited CTC removal was observed, suggesting the weak adsorption/oxidation capacity of N-101-NFF alone. Upon PMS addition, the degradation efficiency increased progressively with increasing PMS dosage and reached 90.3% after 60 min at 0.08 mmol/L. Further increasing the PMS concentration did not provide an obvious improvement, probably because excessive PMS or generated sulfate radicals underwent self-quenching reactions (Equations (1) and (2)). Therefore, 0.08 mmol/L was used as the PMS dosage in the following experiments.

SO_4_•^−^ + SO_4_•^−^ → S_2_O_8_^2−^ (1)

HSO_5_^−^ + SO_4_•^−^ → SO_5_•^−^ + SO_4_^2−^ + H^+^ (2)

#### 2.3.2. Effect of Calcination Temperature

To identify an appropriate pyrolysis temperature, the catalytic performance of samples prepared at different temperatures was evaluated. From the time-dependent degradation profiles in Figure 4c, the CTC degradation efficiencies of the catalysts prepared at pyrolysis temperatures of 600, 700, and 800 °C were 83.8%, 90.3%, and 90.4%, respectively. Although the catalyst pyrolyzed at 800 °C showed a comparable degradation efficiency to that obtained at 700 °C, the improvement was negligible. Therefore, 700 °C was selected as the optimal pyrolysis temperature by considering both catalytic performance and energy consumption.

#### 2.3.3. Influence of Initial pH on CTC Degradation

The initial pH can influence PMS stability, catalyst surface properties, and CTC speciation. Therefore, CTC degradation was examined over a pH range of 3–11 using the N-101-NFF/PMS process. As shown in Figure 4d, acidic conditions were more favorable for CTC removal. This behavior is related to the acid-base dissociation of CTC, which has pK_a_ values of 3.3, 7.4, and 9.3. When the solution pH was below pK_a2_, CTC mainly existed as protonated and neutral/zwitterionic species, including CTCH_3_^+^ and CTCH_2_ [20]. The predominance of these CTC species may favor their enrichment at the catalyst interface, thereby facilitating their contact with PMS-derived reactive species and promoting subsequent oxidative degradation.

#### 2.3.4. Influence of Coexisting Inorganic Anions

**Figure 5:**
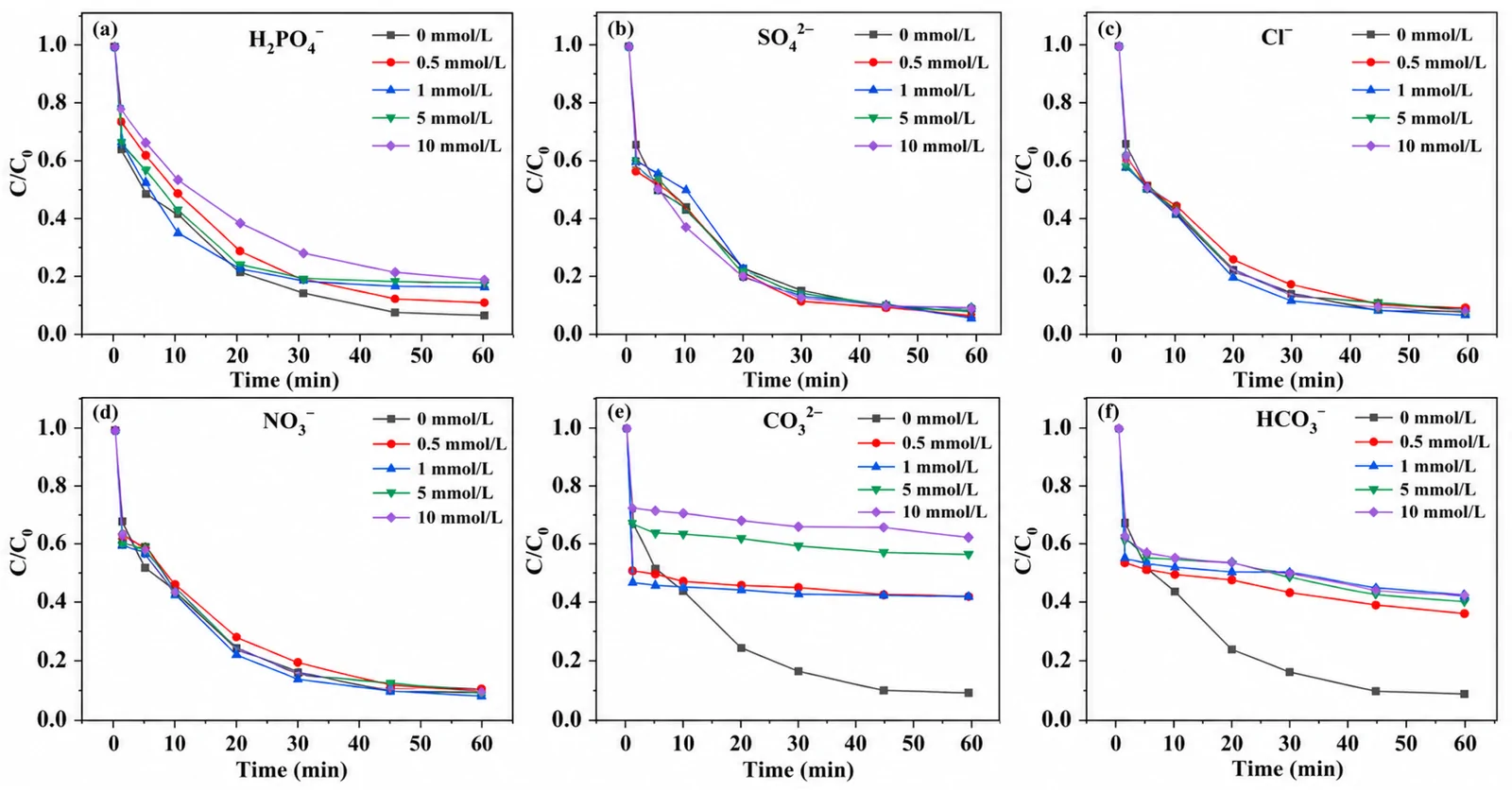
Effects of inorganic ions, including H_2_PO_4_^−^ (**a**), SO_4_^2−^ (**b**), Cl^−^ (**c**), NO_3_^−^ (**d**), CO_3_^2−^ (**e**), and HCO_3_^−^ (**f**), on CTC degradation in the N-101-NFF/PMS system. (CTC = 50 mg/L, PMS = 0.08 mmol/L, pH = 4.86, and N-101-NFF = 1 cm^2^).

The influence of common inorganic anions was evaluated because real water matrices often contain species that can compete for reactive oxygen species or interact with catalyst surfaces. H_2_PO_4_^−^, SO_4_^2−^, Cl^−^, NO_3_^−^, HCO_3_^−^, and CO_3_^2−^ were introduced at concentrations of 0–10 mmol/L. The presence of H_2_PO_4_^−^ inhibits the reaction slightly (Figure 5a), possibly because H_2_PO_4_^−^ consumes free radicals in the solution (Equations (3) and (4)). In addition, H_2_PO_4_^−^ may also form a complex with bivalent metal ions in the catalyst through chelation reaction, occupying part of the active site [21].

SO_4_•^−^+ H_2_PO_4_^−^ → SO_4_^2−^ + H_2_PO_4_• (3)

•OH + H_2_PO_4_^−^ → OH^−^ + H_2_PO_4_• (4)

As shown in Figure 5b–d, SO_4_^2−^, Cl^−^, and NO_3_^−^ show no obvious inhibitory effect. HCO_3_^−^ and CO_3_^2−^ have the most obvious inhibitory effects on the system (Figure 5e,f), and the degradation rates of CTC at the highest concentration (10 mM) are 30.2% and 50.1%, respectively, mainly due to the following three aspects: (1) HCO_3_^−^ and CO_3_^2−^ could react with the main free radicals SO_4_•^−^ and •OH in the PMS system. The number of free radicals is significantly reduced (Equations (5)–(10)) [22]. Compared with highly reactive radicals such as •OH and SO_4_•^−^, the carbonate radical anion (CO_3_•^−^) generally exhibits lower reactivity but higher selectivity toward organic molecules. Its second-order rate constants for reactions with environmentally relevant organic compounds are strongly structure-dependent and can vary over several orders of magnitude [23].

SO_4_•^−^ + HCO_3_^−^ → HCO_3_• + SO_4_^2−^ (5)

•OH + HCO_3_^−^ → CO_3_•^−^ + H_2_O (6)

CO_3_^2−^ +H_2_O → OH^−^ + HCO_3_^−^ (7)

SO_4_•^−^ + CO_3_^2−^ → CO_3_•^−^ + SO_4_^2−^ (8)

•OH + CO_3_^2−^ → CO_3_•^−^ + OH^−^ (9)

HCO_3_• → H^+^ + CO_3_•^−^ (10)

(2) The introduction of CO_3_^2−^ and HCO_3_^−^ may shift the solution toward alkaline conditions, thereby affecting PMS stability and altering the distribution of reactive species. Although alkaline PMS decomposition may promote the formation of O_2_•^−^, •OH, and ^1^O_2_, the simultaneous consumption of SO_4_•^−^/•OH by carbonate species and the generation of less reactive carbonate radicals can weaken the overall oxidation capacity of the system [24]. (3) In addition, CO_3_^2−^/HCO_3_^−^ may coordinate with exposed surface Fe/Ni centers, thereby occupying PMS-activation sites and limiting their accessibility. Such site blocking can hinder PMS adsorption and activation, ultimately weakening the catalytic CTC degradation performance of the N-101-NFF/PMS system [25,26].

From a practical perspective, these results identify carbonate alkalinity as a potential limitation for the application of the N-101-NFF/PMS process to high-alkalinity waters. Elevated HCO_3_^−^ and CO_3_^2−^ concentrations may simultaneously consume SO_4_•^−^/•OH, alter the solution pH and PMS speciation, and reduce the availability of surface Fe/Ni activation sites. Consequently, carbonate-rich matrices may decrease treatment efficiency and require optimization of the PMS dosage, initial pH, or hydraulic residence time to maintain satisfactory CTC removal.

#### 2.3.5. Effect of Water Matrices and Humic Acid on CTC Degradation

To evaluate the water-matrix adaptability of the N-101-NFF/PMS system, CTC degradation was further examined in ultrapure water, tap water, and natural water. The main physicochemical characteristics of these water matrices are summarized in Table S1. As shown in Figure S1a, CTC degradation followed the order ultrapure water (90.3%) > tap water (87.6%) > natural water (70.4%). Although the activity decreased in natural water, the system still maintained a considerable removal efficiency, indicating that N-101-NFF/PMS has tolerance to typical water matrix components. The greater inhibition observed in natural water likely resulted from the combined effects of inorganic ions, natural organic matter, turbidity, and other matrix constituents rather than from a single component. Because the carbonate alkalinity of the tested water samples was not separately quantified, the decrease in activity cannot be attributed specifically to HCO_3_^−^ or CO_3_^2−^. Nevertheless, the controlled anion experiments indicate that carbonate-rich natural waters may present a potential challenge for practical application.

Humic acid (HA), a representative component of natural organic matter, was further introduced to evaluate its effect on CTC degradation in the N-101-NFF/PMS system (Figure S1b). Increasing HA concentration from 0 to 20 mg/L gradually reduced CTC degradation, but the total decrease was only 10.2%. This moderate inhibition suggests that natural organic matter may compete for reactive species or cover active sites, while N-101-NFF/PMS still retains relatively stable activity in the presence of HA.

### 2.4. Stability and Recyclability of N-101-NFF/PMS

The reusability of N-101-NFF was evaluated through consecutive cycling experiments. As shown in Figure S1c, the CTC degradation efficiency decreased only slightly from 90.3% to 88.6% after five consecutive cycles, indicating negligible activity loss. This result demonstrates the favorable recyclability and operational stability of N-101-NFF in PMS-based CTC degradation.

### 2.5. Possible Degradation Mechanism

#### 2.5.1. ROS in N-101-NFF/PMS System

**Figure 6:**
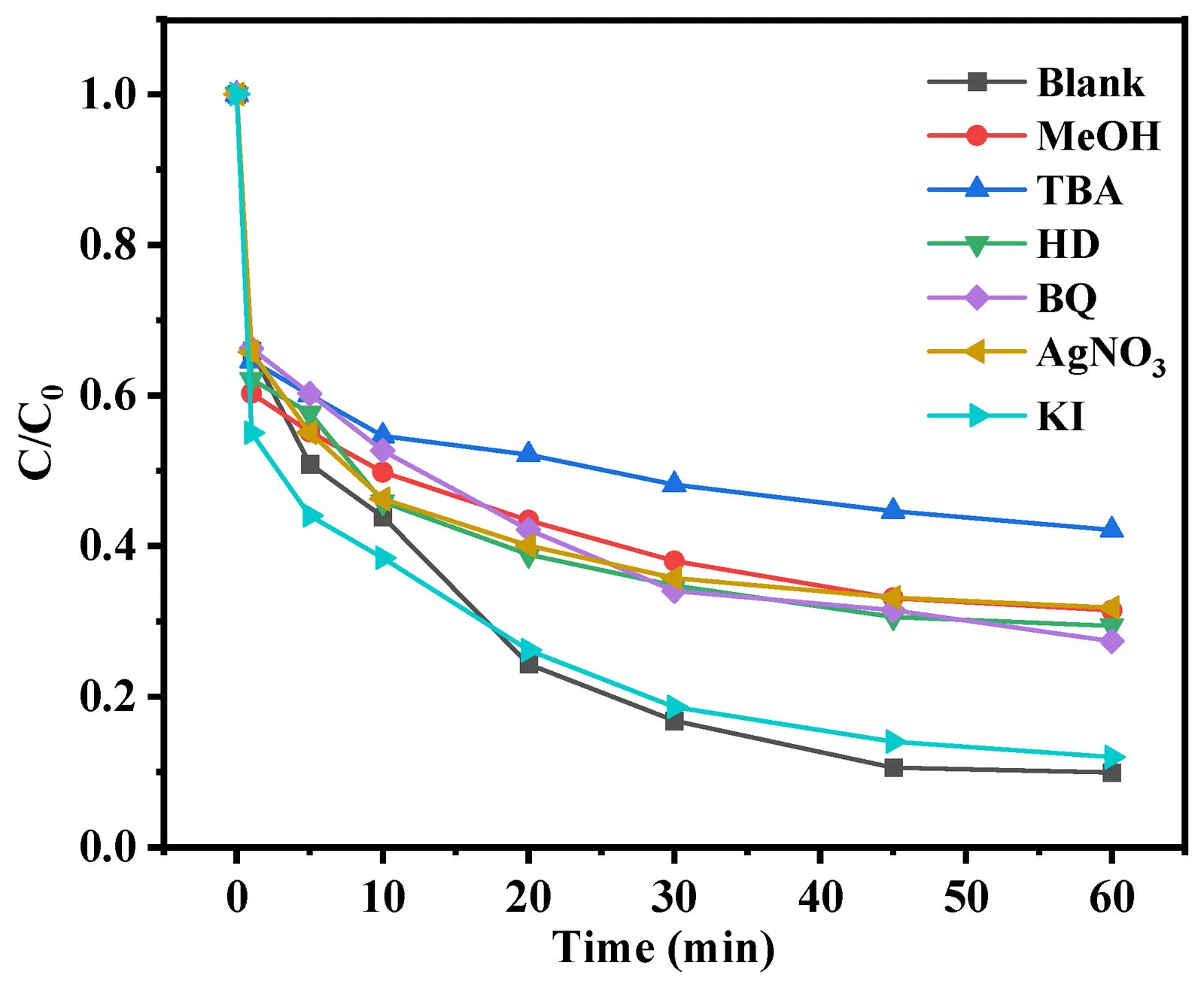
Radical quenching experiments for identifying the reactive species involved in CTC degradation in the N-101-NFF/PMS system.

To clarify the reactive species involved in CTC degradation during N-101-NFF-mediated PMS activation, scavenging experiments were performed using different quenchers, as shown in Figure 6. To distinguish the contributions of different reactive species, MeOH and TBA were selected as alcohol quenching agents for SO_4_•^−^/•OH and •OH, respectively. L-histidine and p-benzoquinone (BQ) were used to probe ^1^O_2_ and O_2_•^−^, while AgNO_3_ and KI were introduced to evaluate the possible involvement of electron-transfer processes and holes, respectively [27].

In the absence of scavengers, the CTC degradation efficiency reached 90.3%. The addition of MeOH, TBA, L-histidine, p-BQ, and AgNO_3_ reduced the CTC degradation efficiencies to 68.8%, 53.6%, 70.8%, 72.9%, and 68.4%, respectively, corresponding to apparent inhibition ratios of 23.8%, 40.6%, 21.6%, 19.3%, and 24.3%. Among the tested scavengers, TBA caused the greatest decrease in CTC degradation, indicating an important contribution from •OH-sensitive oxidation under the tested conditions. The inhibition induced by MeOH, together with the DMPO-EPR signals, further supported the involvement of alcohol-sensitive radical species, including •OH and SO_4_•^−^. Meanwhile, the inhibitory effects of L-histidine and p-BQ were consistent with the participation of ^1^O_2_ and O_2_•^−^, respectively. The decrease observed in the presence of AgNO_3_ suggested the possible involvement of an interfacial electron-transfer process, whereas the negligible effect of KI indicated that hole-mediated oxidation was not a major pathway.

Because the scavengers may exhibit overlapping reactivities and may also influence PMS activation, pollutant–catalyst interactions, and interfacial reactions, the inhibition ratios cannot be directly interpreted as the absolute contributions of individual reactive species. Taken together, the combined quenching and EPR results support the coexistence of radical pathways involving •OH and SO_4_•^−^ and O_2_•^−^ and non-radical pathways involving ^1^O_2_ and interfacial electron transfer in the N-101-NFF/PMS system.

**Figure 7:**
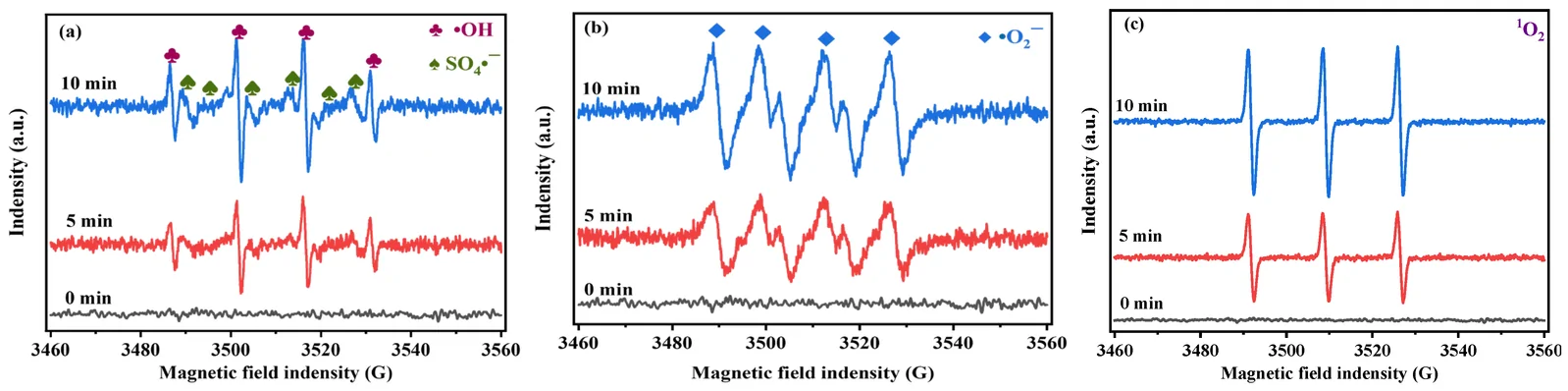
Electron paramagnetic resonance (EPR) spectra obtained using 5,5-dimethyl-1-pyrroline N-oxide (DMPO) for the detection of •OH/SO_4_•^−^ (**a**) and O_2_•^−^ (**b**), and using 2,2,6,6-tetramethylpiperidine (TEMP) for the detection of ^1^O_2_ (**c**) in the N-101-NFF/PMS system.

The reaction components are further validated using EPR tests. DMPO was used to trap •OH/SO_4_•^−^ and O_2_•^−^, while TEMP was used for ^1^O_2_ detection. The quartet signal with an intensity ratio of 1:2:2:1 can be assigned to DMPO-•OH adducts, while weak or overlapped DMPO- SO_4_•^−^ signals may appear near the DMPO-•OH peaks [28]. It is worth noting that the signal strength of •OH in the figure is significantly greater than that of SO_4_•^−^, which may be because SO_4_•^−^ will be rapidly consumed and converted into •OH during the process of CTC degradation. The DMPO spin-trapping spectra showed signals attributable to DMPO-O_2_•^−^ adducts in the N-101-NFF/PMS system (Figure 7b), suggesting the generation of O_2_•^−^ during the reaction [29]. To determine whether ^1^O_2_ is generated, TEMP is used as a spin catcher [30]. According to Figure 7c, a large number of TEMP-^1^O_2_ adduct signals are observed in the N-101-NFF/PMS system. These EPR results demonstrate that N-101-NFF activated PMS to generate •OH, SO_4_•^−^, O_2_•^−^, and ^1^O_2_ which is consistent with the quantitative quenching results and further supports the coexistence of radical and non-radical activation pathways.

#### 2.5.2. Electrochemical Analysis

The inherent electrochemical characteristics of the catalyst are very important for the electron transfer process. In order to further study the electrochemical characteristics of N-101-NFF, the electrochemical characterization of N-101-NFF is carried out. EIS is commonly applied to compare charge-transfer resistance. In the Nyquist diagram shown in Figure S2a, N-101-NFF exhibits a smaller radius than 101-NFF in the high frequency region, indicating that N-101-NFF has a lower resistance. At the same time, a smaller radius generally corresponds to lower interfacial charge-transfer resistance, indicating that N incorporation can improve the electron-transfer efficiency of the material.

CV curves of different catalysts are shown in Figure S2b. The integrated area of the CV curve reflects the redox capacity of the catalyst, and it can be seen that the of N-101-NFF is significantly larger than that of 101-NFF. This shows that N doping can effectively improve the redox ability of the material in the electron transfer pathway. The linear sweep voltammetry (LSV) curve in Figure S2c shows that the addition of both PMS and CTC enhanced the current response, which supports the involvement of interfacial electron transfer in the N-101-NFF/PMS/CTC system. As shown in the i-t curve of Figure S2d, the addition of PMS and CTC successively cause obvious current fluctuations, which is consistent with the conclusion of LSV.

#### 2.5.3. Mechanism of N-101-NFF for PMS Activation

**Figure 8:**
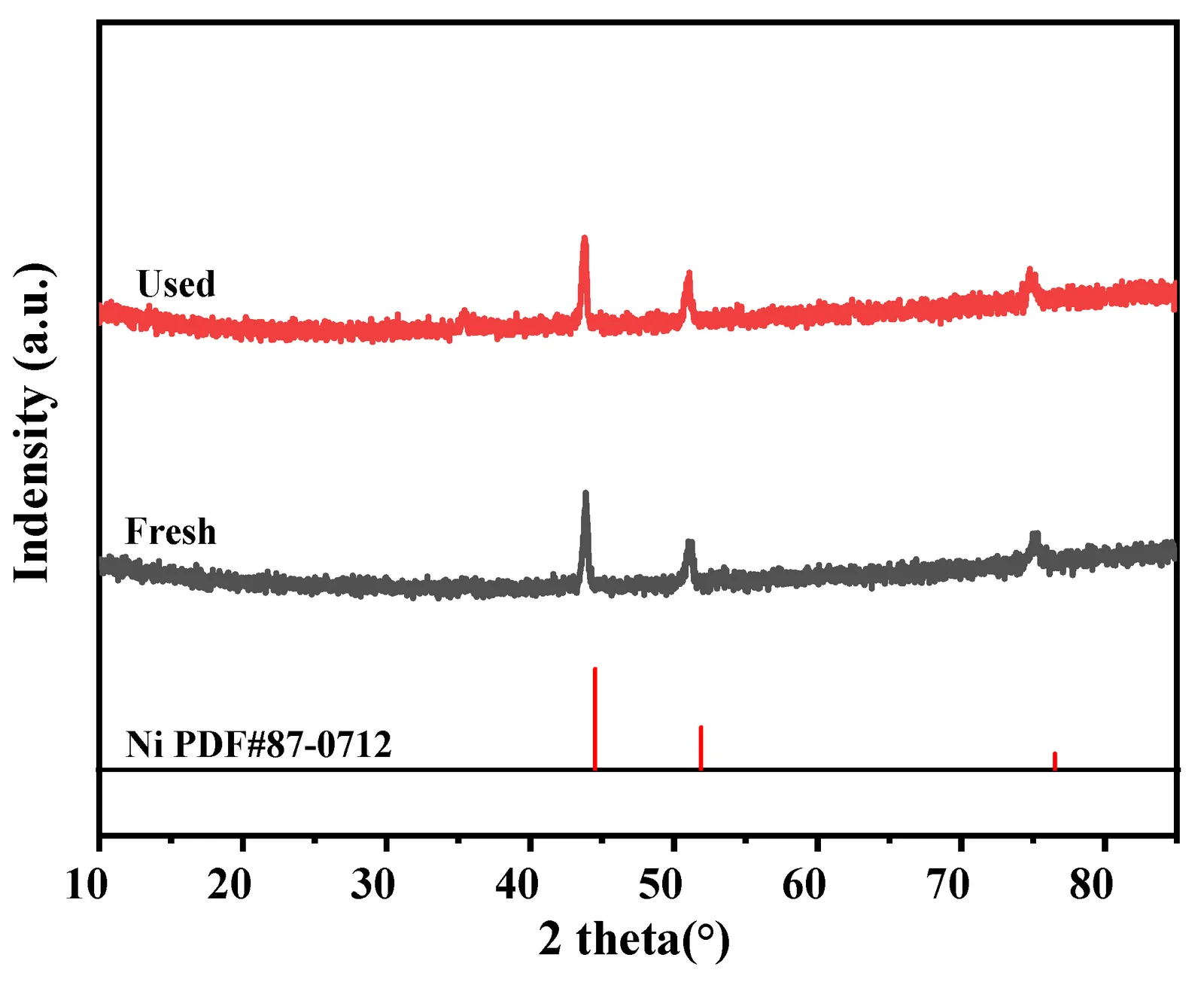
X-ray diffraction (XRD) patterns of fresh and used N-101-NFF before and after CTC degradation.

XRD and XPS analyses were conducted before and after reaction to examine the structural stability and surface chemical evolution of N-101-NFF. As can be seen from XRD measurements (Figure 8), no obvious new characteristic peaks appeared after the reaction, and the intensities of the existing peaks changed only slightly, indicating that the main crystalline features of N-101-NFF were largely retained during PMS activation.

**Figure 9:**
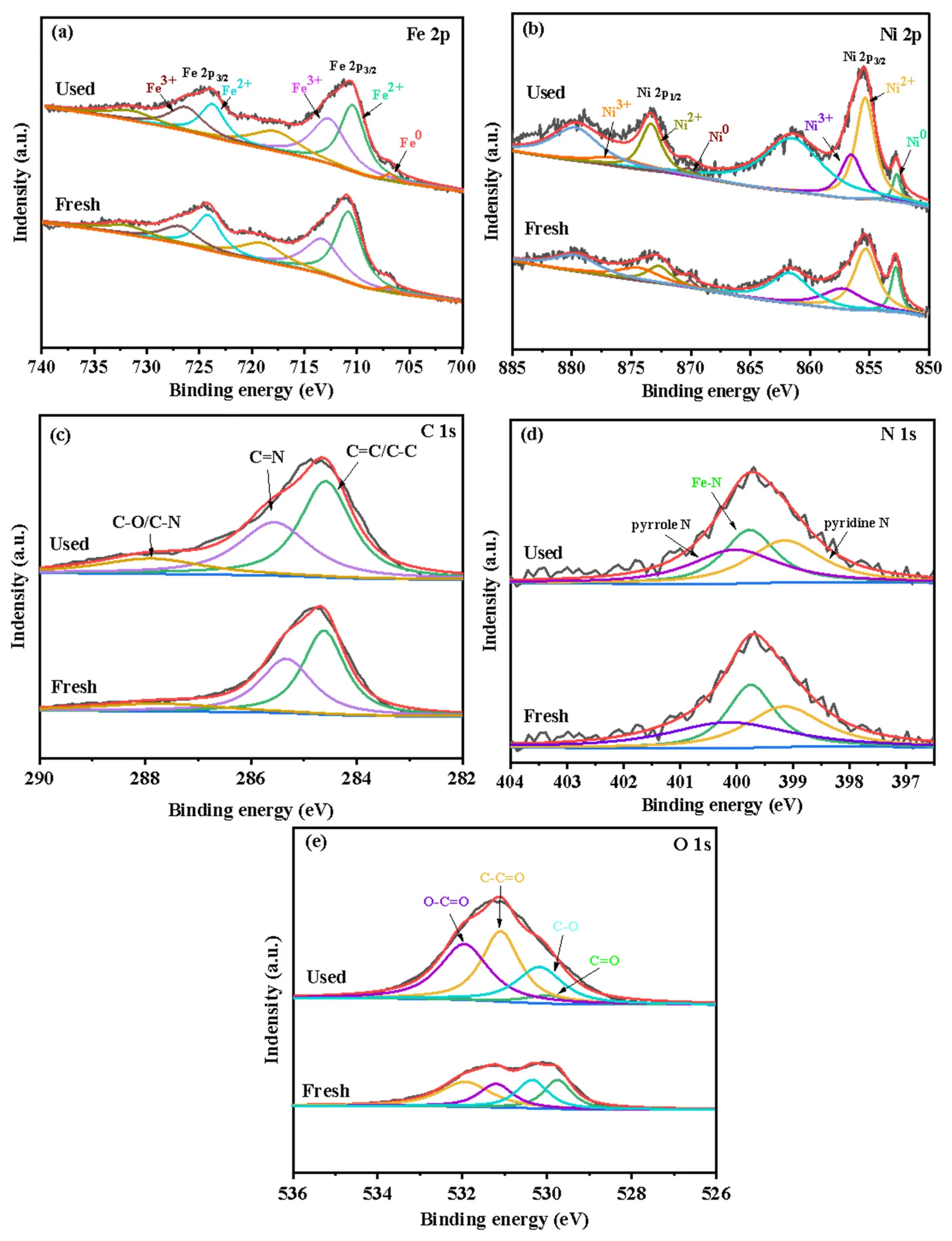
High-resolution XPS spectra of fresh and used N-101-NFF: Fe 2p (**a**), Ni 2p (**b**), C 1s (**c**), N 1s (**d**), and O 1s (**e**).

The Fe 2p high-resolution XPS spectrum (Figure 9a) was deconvoluted to clarify the chemical states of Fe in N-101-NFF. The fitted peaks located at 711.9 and 724.3 eV are assigned to Fe^2+^ 2p_3/2_ and Fe^2+^ 2p_1/2_, respectively, whereas the peaks centered at 713.6 and 727.7 eV correspond to Fe^3+^ species. In addition, the signals observed at 707.5 and 721.3 eV are characteristic of metallic Fe^0^, suggesting the coexistence of Fe^0^, Fe^2+^, and Fe^3+^ in the catalyst. The quantitative fitting results summarized in Table S2 showed that the relative Fe^3+^ content increased from 35.11% before the reaction to 47.48% after the reaction, corresponding to an increase of 12.37 percentage points. In contrast, the Fe^2+^ and Fe^0^ contents decreased from 56.33% to 50.15% and from 8.56% to 2.37%, corresponding to decreases of 6.18 and 6.19 percentage points, respectively. These changes indicate a shift in the surface Fe distribution from the reduced Fe^0^/Fe^2+^ species toward Fe^3+^ after PMS activation, which is consistent with the oxidation of reduced surface Fe species during the reaction.

For the Ni species, the Ni^2+^ content increased from 53.47% to 62.93%, corresponding to an increase of 9.46 percentage points, whereas the Ni^0^ content decreased from 15.94% to 7.16%, corresponding to a decrease of 8.78 percentage points. In comparison, the Ni^3+^ content changed only slightly from 30.59% to 29.91%, corresponding to a decrease of 0.68 percentage points. Therefore, the post-reaction Ni 2p results mainly indicate a decrease in the relative abundance of surface Ni^0^ and a corresponding increase in the relative abundance of Ni^2+^, rather than a pronounced net change in Ni^3+^. These quantitative changes support the participation of Fe- and Ni-containing surface species in PMS activation. However, because XPS was performed on the catalyst after the reaction, the measured values represent net changes in the relative surface valence-state distributions and should not be regarded as direct measurements of the instantaneous Fe^2+^/Fe^3+^ and Ni^2+^/Ni^3+^ distributions during catalysis.

The C 1s spectrum in Figure 9c exhibited three characteristic carbon species with binding energies of 284.71, 285.66, and 287.92 eV, corresponding to C=C/C-C, C=N, and C-O/C-N groups, respectively. Compared with 101-NFF, N-101-NFF exhibited a distinct N 1s signal, confirming the successful introduction of N-containing sites. The N 1s spectra of fresh and used N-101-NFF in Figure 9d were deconvoluted into three components centered at 398.62, 399.73, and 400.48 eV. The signals at 398.62 and 400.48 eV were assigned to pyridinic N and pyrrolic N, respectively, which may regulate the local electronic environment and provide potential sites for PMS activation [31], whereas the signal at 399.73 eV was attributed to Fe-N coordination, which may facilitate electron exchange between the catalyst and PMS [32,33]. After PMS activation, the relative contents of pyridinic N, Fe-N, and pyrrolic N changed from 34.74%, 31.61%, and 33.65% to 33.56%, 32.47%, and 33.97%, corresponding to changes of -1.18, +0.86, and +0.32 percentage points, respectively. These relatively small variations suggest that the principal N-containing structures were largely retained during the reaction, while their slight redistribution may be associated with interfacial electronic interactions.

The O 1s spectrum in Figure 9e was deconvoluted into four fitted components at 529.86, 530.42, 531.23, and 532.23 eV, which were assigned to C=O, C-O, C-C=O, and O-C=O, respectively. Among the two oxygen-containing components quantified in Table S2, the relative area assigned to C=O decreased from 21.36% to 4.82%, corresponding to a decrease of 16.54 percentage points, whereas that assigned to C-O decreased from 23.71% to 22.49%, corresponding to a decrease of 1.22 percentage points. The more pronounced variation in the C=O-related component suggests that carbonyl-related surface species may participate in PMS activation and the associated non-radical oxidation process [34]. Carbonyl-containing sites have also been reported to facilitate electron-transfer-associated persulfate activation [35].

Combined with the smaller EIS semicircle and larger integrated CV area of N-101-NFF relative to 101-NFF, these results suggest that N-containing and carbonyl-related sites may play complementary roles in PMS activation. N-containing sites may regulate the local electronic structure and facilitate interfacial charge transfer, whereas carbonyl-related sites may provide additional interaction sites for PMS. Their coexistence may contribute to both Fe/Ni-mediated radical generation and electron-transfer-associated non-radical pathways. Nevertheless, the present XPS and electrochemical results do not permit quantitative separation of the individual contributions of the two types of sites.

**Figure 10:**
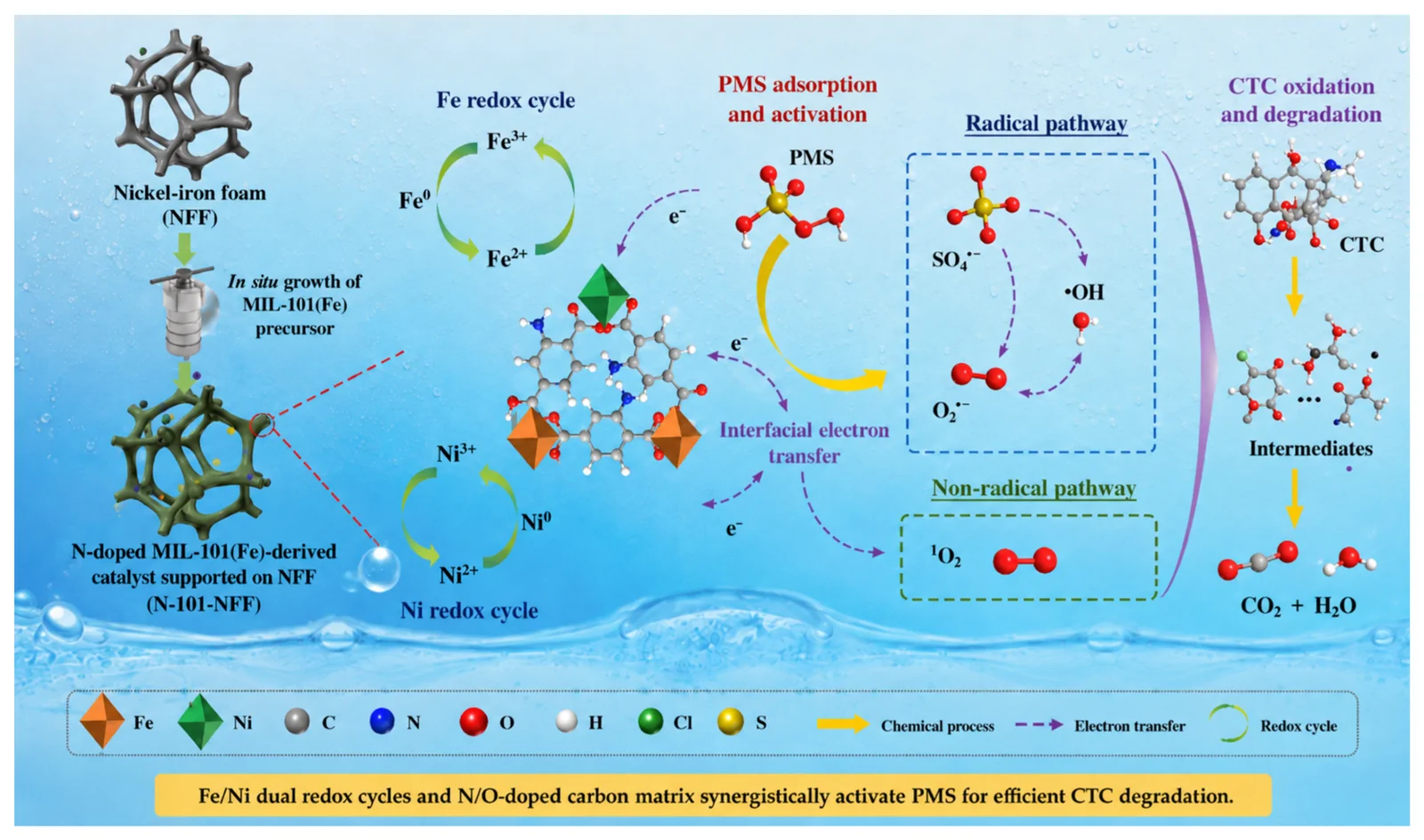
Mechanism diagram of N-101-NFF/PMS system for degradation of CTC.

According to the previous experimental conclusions, the possible reaction process of N-101-NFF/PMS [36,37] (Equations (11)–(24)) and the degradation mechanism of CTC are proposed (Figure 10).

Fe^0^/Ni^0^ + 2 HSO_5_^−^ → Fe^2+^/Ni^2+^ + 2SO_4_•^−^ + 2OH^−^ (11)

Fe^2+^/Ni^2+^ + HSO_5_^−^ → Fe^3+^/Ni^3+^ + SO_4_•^−^ + OH^−^ (12)

SO_4_•^−^ + H_2_O → SO_4_^2−^ + •OH + H^+^ (13)

Fe^3+^/Ni^3+^ + HSO_5_^−^ → Fe^2+^/Ni^2+^ + SO_5_•^−^ + H^+^ (14)

Fe^3+^/Ni^3+^ + 2 SO_5_•^−^ → Fe^2+^/Ni^2+^ + 2 SO_4_•^−^ + O_2_ (15)

HSO_5_^−^ → SO_5_^2−^ + H^+^ (16)

SO_5_^2−^+H_2_O → H_2_O_2_+ SO_4_^2−^ (17)

•OH + H_2_O_2_ → HO_2_• + H_2_O (18)

HO_2_• → O_2_•^−^ + H^+^ (19)

Fe^0^/Ni^0^ + 2 Fe^3+^/Ni^3+^ → 3 Fe^2+^/Ni^2+^ (20)

2 SO_5_•^−^ + H_2_O → 2 HSO_4_^−^ + 1.5 ^1^O_2_ (21)

2 Fe^2+^/Ni^2+^ + 2 SO_5_•^−^ → 2 Fe^3+^/Ni^3+^ + 2 SO_4_•^−^ + ^1^O_2_ (22)

HSO_5_^−^ +SO_5_^2−^ → HSO_4_^−^ + SO_4_^2−^ + ^1^O_2_ (23)

HSO_5_^−^ + C=O → C=O + SO_4_^2−^ + ^1^O_2_ (24)

In the radical pathway, Fe-N coordination structures can act as electron-regulating centers because of their ability to draw electrons from adjacent atoms [38,39]. In contrast, the carbon atoms neighboring the Fe-N coordination structure are relatively unfavorable for electron loss, suggesting their weaker electron-donating capability. Therefore, the Fe-N coordination sites can facilitate interfacial electron transfer between the catalyst and adsorbed PMS, thereby promoting PMS activation and the generation of reactive oxygen species [40,41,42]. In particular, PMS can be activated by Fe^0^/Ni^0^ to produce SO_4_•^−^ by electron transfer (Equation (11)). In addition, Fe^2+^/Ni^2+^ can also effectively activate PMS to produce SO_4_•^−^ (Equation (12)). The resulting SO_4_•^−^ is then further transformed into •OH by (Equation (13)). Fe^3+^/Ni^3+^ can also effectively catalyze PMS to produce SO_5_•^−^, SO_5_•^−^ which is further catalyzed by Fe^3+^/Ni^3+^ to produce SO_4_•^−^ (Equations (14) and (15)). In addition, the naturally hydrolyzed products of PMS react with the previously produced •OH to form O_2_•^−^ and H^+^ (Equations (16)–(19)). In addition, the presence of zero-valent metals can effectively improve the Fe^3+^/Ni^3+^/Fe^2+^/Ni^2+^ cycle and improve the catalytic effect of PMS (Equation (20)).

For non-free radical processes, PMS is adsorbed to the surface of the catalyst to react with Fe^3+^ or Ni^2+^ to form SO_5_•^−^, which contributes to the formation of ^1^O_2_, and the automatic decomposition of PMS can also produce ^1^O_2_ (Equations (21)–(23)). Moreover, carbonyl-related oxygenated groups may participate in PMS adsorption and interfacial electron transfer, thereby contributing to PMS activation and ^1^O_2_ generation (Equation (24)). Based on the quantitative quenching experiments and EPR results, CTC degradation in the N-101-NFF/PMS system involved coexisting radical and non-radical pathways. Among the tested scavengers, TBA produced the strongest apparent inhibition, indicating an important contribution from •OH-sensitive oxidation. Meanwhile, the combined quenching and EPR results supported the participation of SO_4_•^−^, O_2_•^−^, and ^1^O_2_, while the AgNO_3_ inhibition and electrochemical responses suggested the possible involvement of interfacial electron transfer.

#### 2.5.4. CTC Degradation Products/Pathways

**Figure 11:**
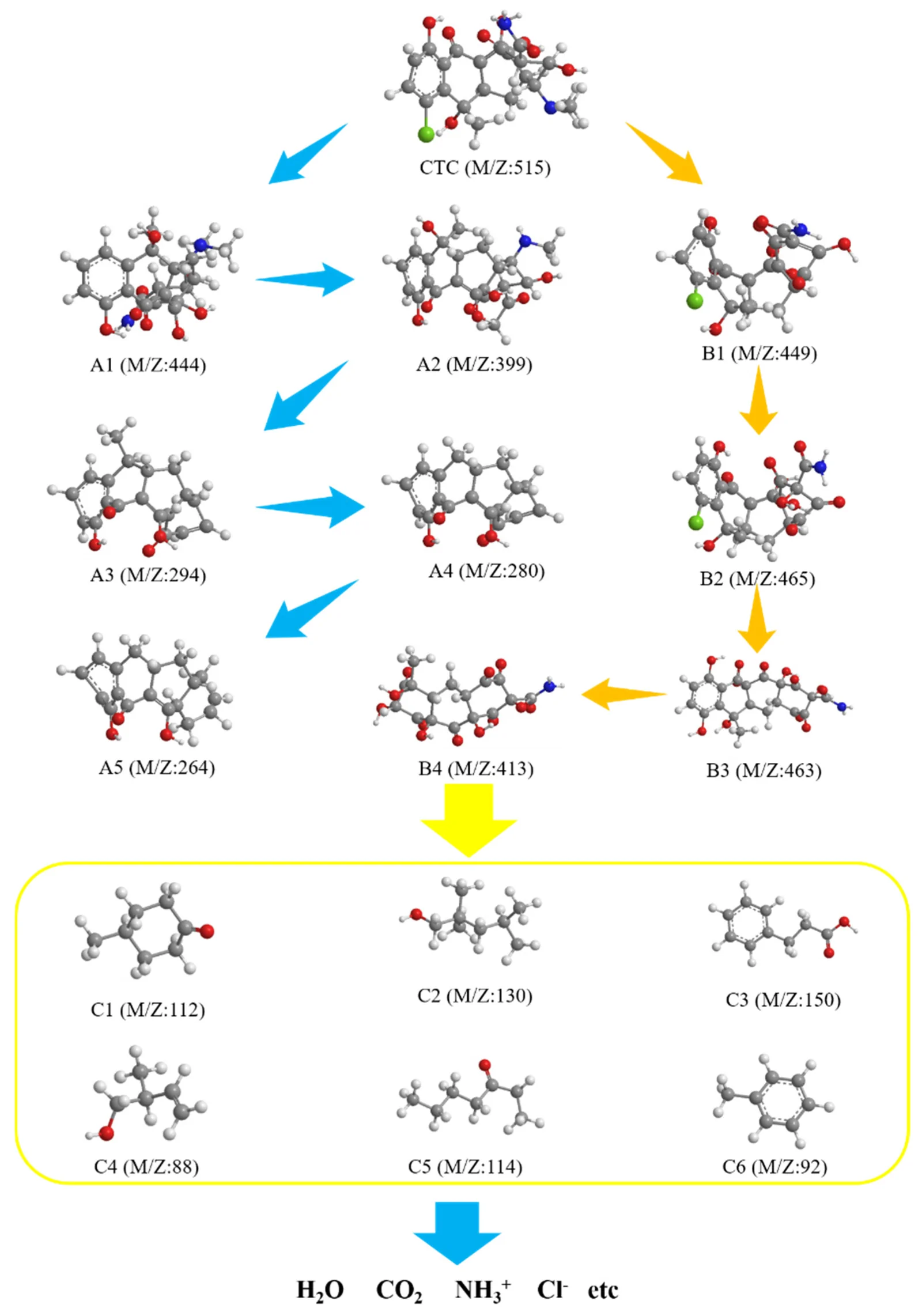
Possible degradation path of CTC in N-101-NFF/PMS system.

LC-MS was employed to identify the transformation intermediates formed during CTC degradation in the N-101-NFF/PMS system. The LC-MS fragmentation data and proposed structures of the identified intermediates are summarized in Table S3. The results showed that 15 major intermediates with mass-charge ratios (*m*/*z*) of 444, 399, 294, 280, 264, 449, 465, 463, 413, 112, 130, 150, 88, 114, and 92 are identified. Two possible CTC degradation pathways were shown in Figure 11. For pathway I: First, under the attack of SO_4_•^−^ and •OH, CTC molecules dechlorinate to form A1 (*m*/*z* = 444) [43]. Subsequently, the transformation from A1 to A2 (*m*/*z* = 399) was tentatively assigned to demethylation and deamination. Other intermediates may have originated from successive transformations of the CTC skeleton, including demethylation, deamination, dehydroxylation/hydroxyl substitution, loss of carbonyl- or formyl-containing groups, C=C bond cleavage, and ring-opening reactions [44,45]. For pathway II, the tertiary amine moiety of CTC is attacked by •OH to form B1 (*m*/*z* = 449). At the same time, the attack of •OH causes the ketone group in B1 to be replaced, thus forming the intermediate B2 (*m*/*z* = 465). Then, B2 is converted to B3 (*m*/*z* = 463) by dechlorination and hydroxyl substitution. B3 was converted to B4 (*m*/*z* = 413) through ring-opening reactions [46,47]. With prolonged oxidation, intermediates from both pathways may be further fragmented into low-molecular-weight organic compounds and potentially converted into CO_2_, H_2_O, Cl^−^, and inorganic nitrogen-containing species.

#### 2.5.5. Toxicity Analysis of Degradation Intermediates

**Figure 12:**
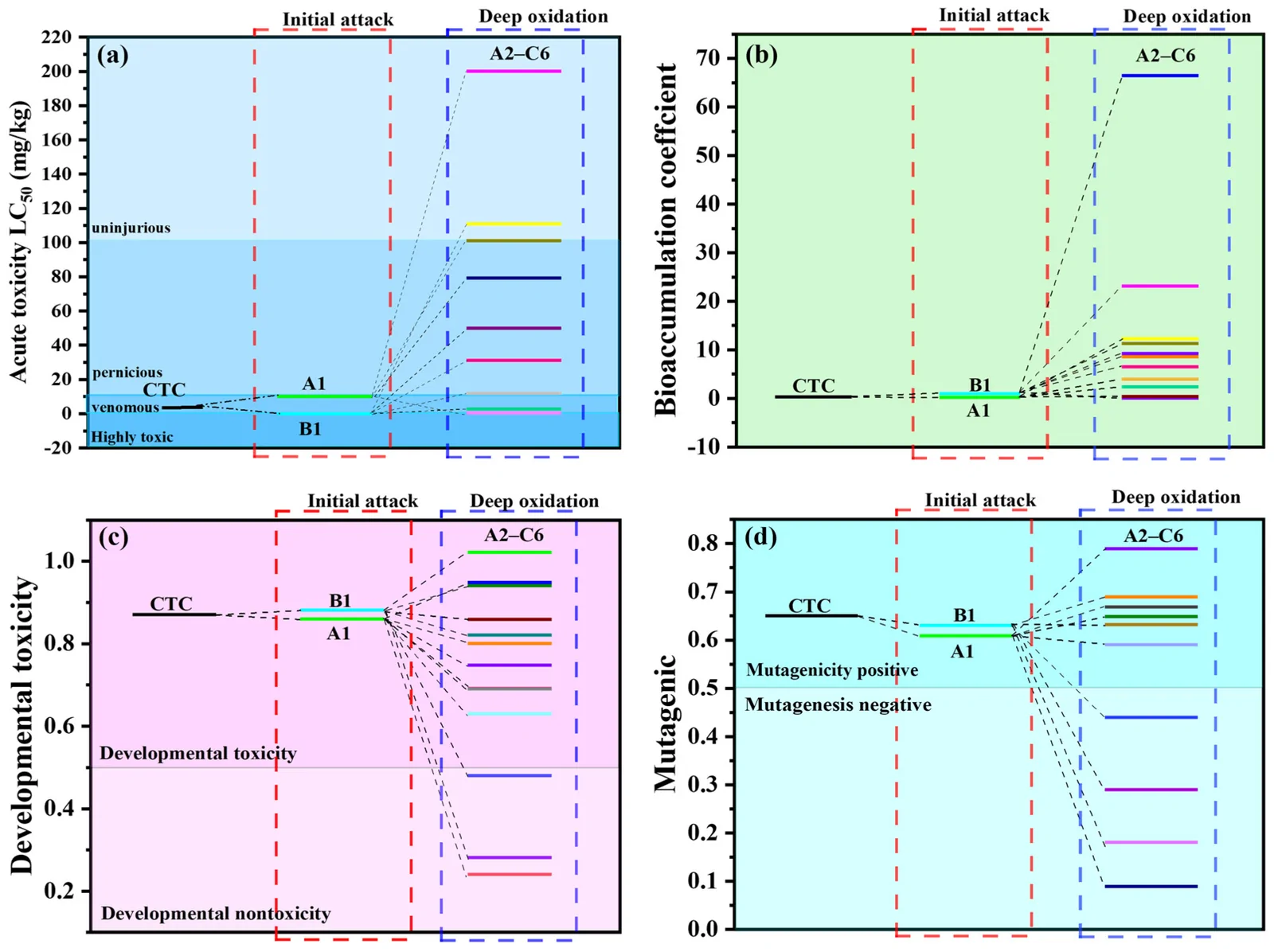
Predicted acute toxicity (**a**), bioaccumulation factor (**b**), developmental toxicity (**c**), and mutagenicity (**d**) of CTC and its degradation intermediates in the N-101-NFF/PMS system.

The potential toxicity of CTC and its degradation intermediates was predicted using the Toxicity Estimation Software Tool (T.E.S.T., version 5.1.2, U.S. Environmental Protection Agency, Washington, DC, USA), with the corresponding results summarized in Table S3. LC_50_ refers to median lethal concentration for fathead minnows in a given period of time, and in general, the lower the LC_50_ value, the more toxic it is [48]. As shown in Figure 12a, some intermediates, such as A3–A5 and B1, exhibited lower LC_50_ values than CTC, indicating temporarily increased acute toxicity during the initial degradation stage. In addition, however, small-molecule products generated during deep oxidation showed much higher LC_50_ values, suggesting reduced acute toxicity after further degradation. Therefore, the N-101-NFF/PMS-mediated oxidation process can effectively reduce the overall toxicity of CTC. As shown in Figure 12b, several transformation intermediates exhibited higher bioaccumulation factors than CTC, suggesting that these products may possess an increased tendency for environmental enrichment and persistence. Figure 12c compares the predicted developmental toxicity of CTC with that of its transformation intermediates. The developmental toxicity of most intermediates has decreased, and some of them have decreased to no toxicity. For mutagenicity (Figure 12d), the same trend as for developmental toxicity is observed, with most intermediates lower than CTC and some reaching mutagenicity negative. Overall, although several CTC transformation intermediates exhibited increased bioaccumulation potential, their predicted acute toxicity, developmental toxicity, and mutagenicity generally decreased after further oxidation. These results suggest that the N-101-NFF/PMS oxidation process may reduce the overall predicted toxicity associated with CTC transformation products, provided that sufficient oxidation is achieved. However, the temporary formation of intermediates with higher acute toxicity or bioaccumulation potential suggests that incomplete degradation products should not be ignored. Therefore, sufficient reaction time and further mineralization are necessary to minimize the potential ecological risks associated with transient transformation products [49,50,51].

### 2.6. Fixed Bed Applications for N-101-NFF/PMS Systems

**Figure 13:**
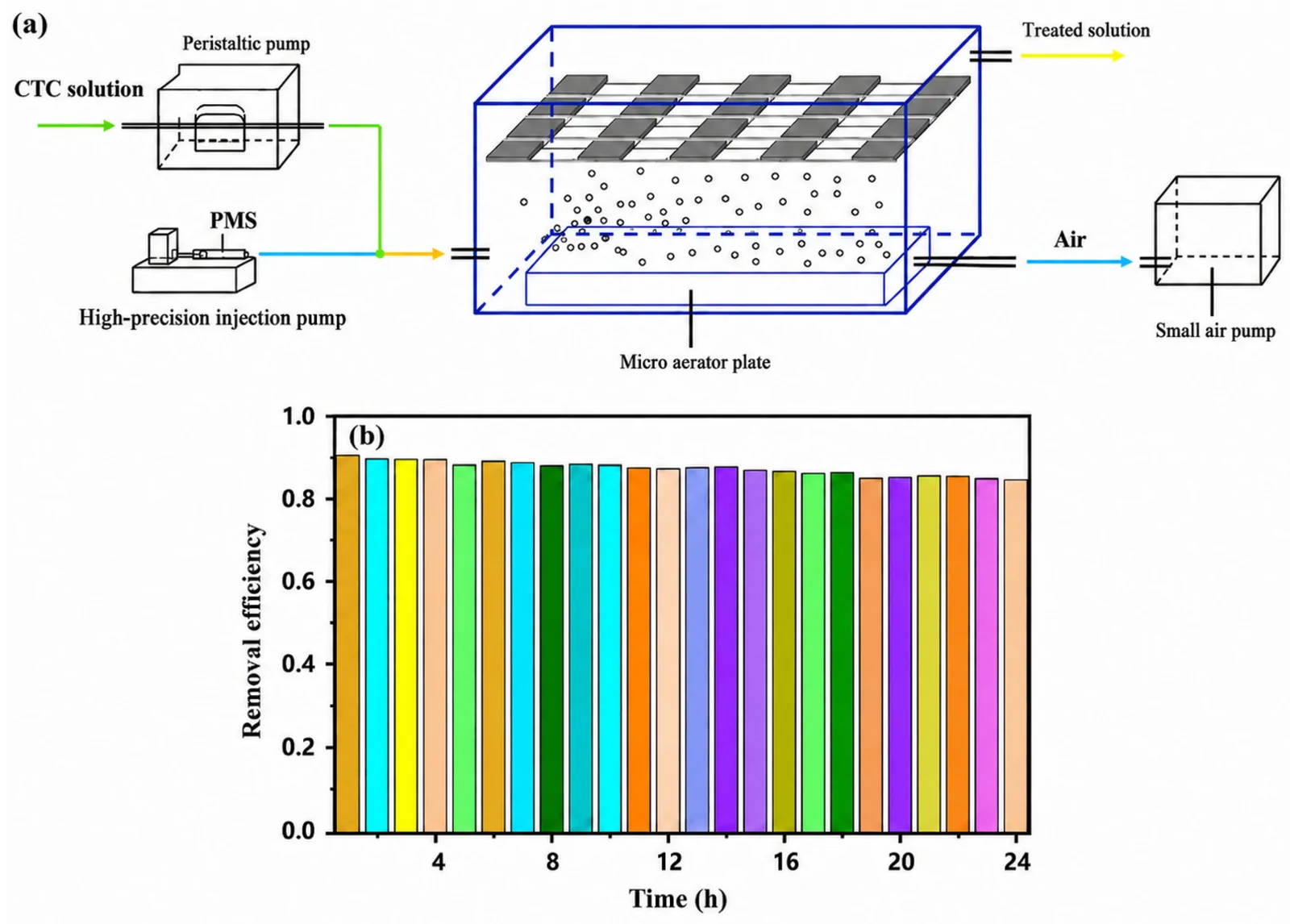
Continuous-flow evaluation of the N-101-NFF/PMS fixed-bed reactor: (**a**) reactor configuration and (**b**) time-dependent CTC removal over 24 h. Operating conditions: initial CTC concentration = 50 mg/L, CTC feed rate = 200 mL/h, PMS feed concentration = 0.1 mol/L, PMS dosing rate = 200 μL/h, and effective N-101-NFF area = 100 cm^2^.

To evaluate the continuous-flow applicability of the N-101-NFF/PMS system, a fixed-bed reactor was constructed using N-101-NFF as the catalytic bed (Figure 13a), where the CTC feed solution and PMS stock solution were continuously fed at flow rates of 0.2 L/h and 200 μL/h, respectively. As shown in Figure 13b, the CTC removal efficiency slightly decreased during 24 h of operation but remained above 86%, indicating the stable catalytic performance of N-101-NFF under fixed-bed conditions.

Compared with conventional powder MOF-derived catalysts, which are dispersed in the reaction solution and generally require filtration, centrifugation, or an additional immobilization step for recovery and continuous use, N-101-NFF integrates the MOF-derived active phase with a macroscopic nickel–iron foam substrate. This monolithic configuration enables direct catalyst recovery and stable placement as a fixed catalytic bed, while reducing the risk of catalyst washout and avoiding an additional solid–liquid separation step after treatment. Therefore, the continuous-flow result demonstrates not only the catalytic stability of N-101-NFF but also its operational advantage for catalyst retention and continuous treatment. Nevertheless, the present 24 h experiment should be considered a proof-of-concept evaluation, and longer-term operation in actual water matrices is required to assess possible activity loss associated with matrix interference, surface fouling, and carbonate inhibition.

### 2.7. Comparison with Previously Reported Tetracycline-Class Antibiotic Degradation Systems

A direct comparison was made with the recently reported Co-doped N-101-NFF(Co_0.05_) catalyst because the two systems were prepared using closely related MOF-derived monolithic strategies. Both catalysts were obtained through the in situ growth of an amino-functionalized Fe-based MOF precursor on nickel–iron foam followed by pyrolysis, whereas the previously reported synthesis additionally included a Co precursor to construct a Co-containing Fe/Ni-based monolithic catalyst. Under comparable batch conditions, including 50 mg/L CTC, 0.08 mmol/L PMS, and an effective catalyst area of 1 cm^2^, N-101-NFF and N-101-NFF(Co_0.05_) achieved CTC degradation efficiencies of 90.3% and 95.7%, respectively, within 60 min. These results indicate that Co incorporation was associated with higher catalytic activity, enhanced Fe/Ni/Co redox interactions, and a greater contribution from the ^1^O_2_^−^mediated non-radical pathway, whereas the present N-101-NFF/PMS system involved both radical and non-radical pathways, with ·OH making an important contribution.

The present synthesis avoids the intentional addition of Co and retains a relatively simple binary Fe/Ni composition, while the in situ growth strategy directly integrates the active phase with a recoverable monolithic substrate suitable for fixed-bed operation. Nevertheless, the use of DMF, a 24 h solvothermal treatment, and pyrolysis at 700 °C for 3 h may increase solvent use and energy demand during scale-up. The catalytic activity was also moderately lower than that of the Co-doped derivative, and continuous-flow performance has thus far been evaluated over only 24 h. To further compare the monolithic configuration with conventional MOF-derived powder catalysts, Fe-NPC-600/PMS and Co@N-MC/PMS were included as representative systems for TC degradation. Fe-NPC-600 achieved approximately 90% TC removal within 20 min, whereas Co@N-MC achieved 91.27% TC degradation within 30 min. Although these powder catalysts exhibited batch degradation efficiencies comparable to that of N-101-NFF, their dispersed configurations require subsequent magnetic recovery or solid–liquid separation, and continuous-flow performance was not reported in the cited studies. In contrast, N-101-NFF can be directly retained as a fixed catalytic bed and maintained more than 86% CTC removal during 24 h of continuous-flow operation. Because the target antibiotics, catalyst dosages, PMS dosages, and operating conditions differed among the reported studies, the comparison in Table 1 is intended to provide contextual information rather than a direct ranking of intrinsic catalytic activity.

## 3. Materials and Methods

### 3.1. Material and Chemicals

Ferric chloride hexahydrate (FeCl_3_·6H_2_O), nickel chloride hexahydrate (NiCl_2_·6H_2_O), terephthalic acid (H_2_BDC), 2-aminoterephthalic acid (NH_2_-H_2_BDC), *N*,*N*-dimethylformamide (DMF), ethanol, methanol, nitric acid (HNO_3_), sulfuric acid (H_2_SO_4_), sodium hydroxide (NaOH), potassium chloride (KCl), silver nitrate (AgNO_3_), tert-butanol (TBA), p-benzoquinone (p-BQ), sodium oxalate (Na_2_C_2_O_4_), potassium iodide (KI), L-histidine, potassium dihydrogen phosphate (KH_2_PO_4_), sodium sulfate (Na_2_SO_4_), sodium chloride (NaCl), potassium nitrate (KNO_3_), sodium carbonate (Na_2_CO_3_), and sodium bicarbonate (NaHCO_3_) were supplied by Sinopharm Chemical Reagent Co., Ltd. (Shanghai, China). Chlortetracycline hydrochloride (CTC, C_22_H_23_ClN_2_O_8_·HCl) and potassium peroxymonosulfate (PMS, 47% as KHSO_5_) were purchased from Aladdin Reagent Co., Ltd. (Shanghai, China). Spectroscopic-grade potassium bromide (KBr) was obtained from Thermo Fisher Scientific (Waltham, MA, USA). Commercial nickel foam (NF) and nickel–iron foam (NFF) were purchased from Kunshan Xingzhenghong Electronic Materials Co., Ltd. (Kunshan, China) and employed as monolithic substrates. Unless otherwise stated, all chemicals were of analytical grade and used as received. Deionized water was used for preparing all aqueous solutions.

### 3.2. Synthesis of Catalysts

#### 3.2.1. Pretreatment of Nickel–Iron Foam and Nickel Foam

NFF or NF was first treated in 4 mol/L HCl under ultrasonication for 3 min to remove surface impurities and oxides. The foams were subsequently rinsed with ethanol and deionized water under identical ultrasonic conditions. The cleaned substrates were then dried at 60 °C before further use.

#### 3.2.2. Synthesis of 101-NF and 101-NFF

101-NF and 101-NFF were prepared through an in situ solvothermal growth strategy followed by high-temperature thermal treatment. Typically, H_2_BDC (5.0 mmol) and FeCl_3_·6H_2_O (10 mmol) were separately dissolved in DMF (30 mL each) under stirring to form homogeneous ligand and metal precursor solutions, respectively. The two solutions were then combined, and the pretreated NF or NFF substrate was immersed in the resulting reaction mixture. After further stirring to facilitate the uniform infiltration of precursors and their sufficient interaction with the foam framework, the mixture was sealed in a Teflon-lined autoclave and maintained at 110 °C for 24 h. After the solvothermal treatment, the autoclave was allowed to cool naturally to room temperature. The resulting products were washed repeatedly with ethanol and deionized water to remove residual precursors and then dried overnight at 80 °C. Subsequently, the dried intermediates were pyrolyzed in a tube furnace at 700 °C for 3 h with a heating ramp of 5 °C min^−1^, yielding 101-NF and 101-NFF.

#### 3.2.3. Synthesis of N-101-NFF

**Figure 14:**
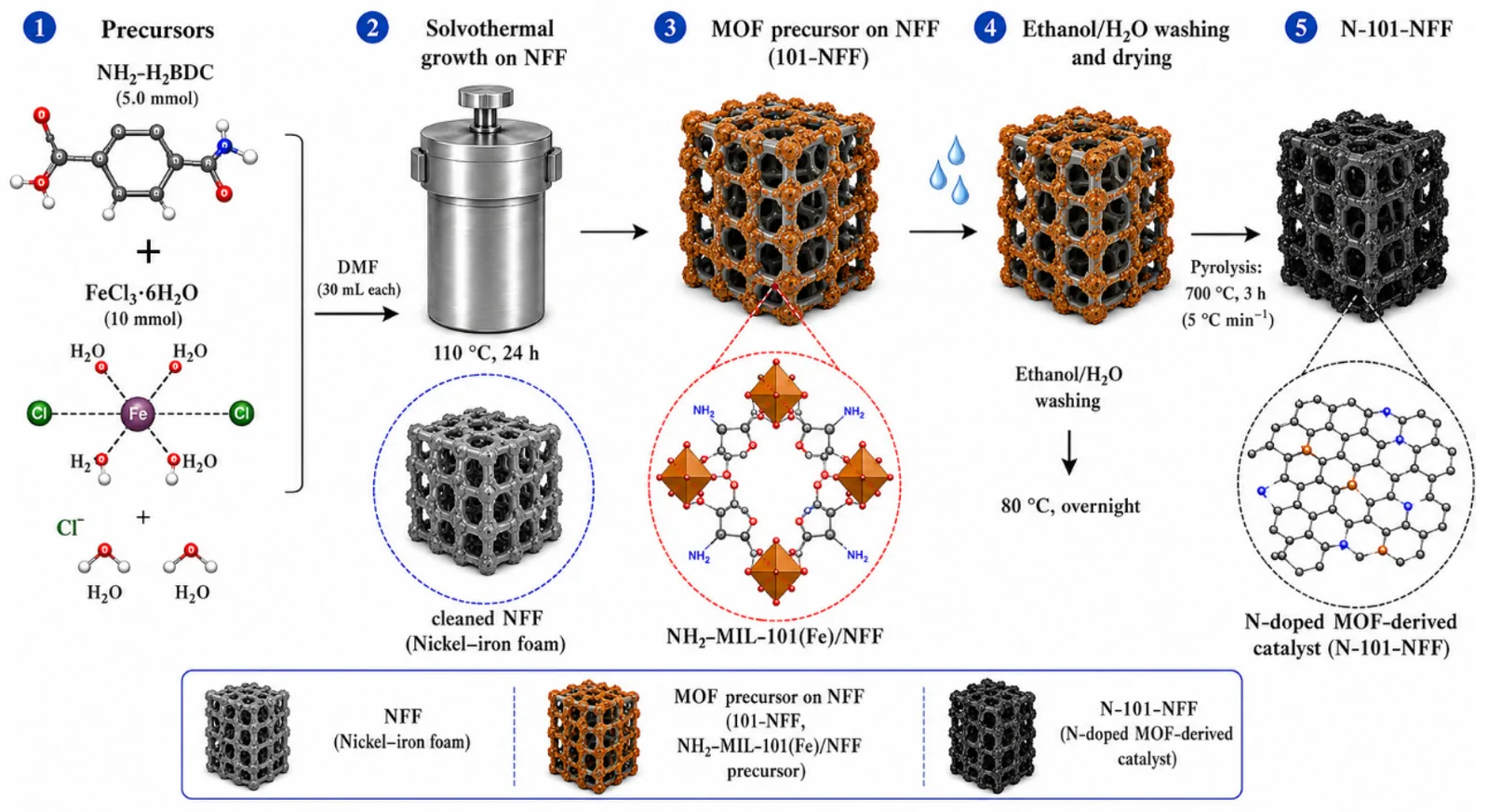
The synthesis process of N-101-NFF.

N-101-NFF was synthesized using NH_2_-H_2_BDC as the N-containing organic ligand. Briefly, NH_2_-H_2_BDC (5.0 mmol) and FeCl_3_·6H_2_O (10 mmol) were separately dissolved in DMF (30 mL each) and stirred until clear precursor solutions were formed. The two solutions were then combined, followed by immersion of the cleaned NFF substrate. After the mixture was stirred until homogeneous, it was transferred into a Teflon-lined autoclave and maintained at 110 °C for 24 h. The obtained product was washed repeatedly with ethanol and deionized water to remove residual precursors, and then dried overnight at 80 °C. Subsequently, the dried sample was pyrolyzed at 700 °C for 3 h with a heating ramp of 5 °C min^−1^. The synthetic procedure for N-101-NFF is schematically presented in Figure 14.

### 3.3. Characterization of Catalysts

(Supporting Information, Text S1).

### 3.4. Experimental Methods

For each batch degradation test, 50 mL of CTC solution (50 mg/L) was used as the reaction medium. A piece of N-101-NFF with an effective catalytic area of 1 cm^2^ was placed in the solution, followed by the addition of PMS to obtain a final concentration of 0.08 mmol/L. The mixture was continuously agitated at room temperature throughout the reaction. At predefined time points, 1 mL samples were withdrawn with disposable syringes, filtered through 0.22 μm polyethersulfone membranes, and diluted five times prior to measurement. The remaining CTC concentration was quantified by monitoring the absorbance at 365 nm using a UV–Vis spectrophotometer (UV-2600, Shimadzu Corporation, Kyoto, Japan).

The degradation efficiency of CTC was calculated based on Equation (25):

Degradation efficiency (%) = (1 − C_t_/C_0_) × 100% = (1 − A_t_/A_0_) × 100% (25)

where C_0_ and Ct denote the CTC concentrations before reaction and at reaction time t, respectively. A_0_ and At are the corresponding absorbance values measured at 365 nm. Within the linear absorbance range, the Beer-Lambert relationship allows C_t_/C_0_ to be approximated by A_t_/A_0_.

Fixed-bed experiment: Continuous-flow tests were conducted in a fixed-bed reactor consisting of a CTC feed line, a PMS dosing line, a N-101-NFF catalytic bed, and a bottom-mounted microbubble aeration unit. During operation, 20 L of CTC solution (50 mg/L) was delivered into the reactor at 0.2 L/h using a peristaltic pump, while PMS stock solution (0.1 mol/L) was continuously injected at 200 μL/h using a high-precision injection pump. N-101-NFF with an effective area of 100 cm^2^ was positioned in the reactor as the catalytic bed. The microbubble aeration unit was used to enhance liquid-phase mass transfer. Effluent samples were collected at 1 h intervals, filtered through 0.45 μm membranes, diluted fivefold, and quantified by UV-vis spectroscopy.

## 4. Conclusions

This study developed a *N*-doped monolithic Fe/Ni-based catalyst, N-101-NFF, through in situ growth on nickel–iron foam followed by pyrolysis, and applied it for PMS activation toward CTC degradation in aqueous solution. Benefiting from its monolithic structure, Fe/Ni redox sites, N-containing active sites, and enhanced electron-transfer ability, N-101-NFF exhibited efficient PMS activation and achieved 90.3% CTC degradation within 60 min. The N-101-NFF/PMS system showed better degradation performance under acidic conditions and maintained effective activity in different water matrices. Quenching experiments and EPR analysis revealed the coexistence of radical and non-radical oxidation pathways involving SO_4_•^−^, •OH, O_2_•^−^, and ^1^O_2_, with •OH-sensitive oxidation showing the strongest apparent response in the scavenging experiments. The Fe^2+^/Fe^3+^ and Ni^2+^/Ni^3+^ redox cycles, together with Fe^0^/Ni^0^, Fe-N, pyridinic N, pyrrolic N, C=N, and C=O sites, jointly contributed to PMS activation. Moreover, N-101-NFF exhibited favorable durability and recyclability, with the CTC degradation efficiency decreasing only slightly from 90.3% to 88.6% after five cycles. Continuous-flow experiments further demonstrated that the monolithic catalyst maintained more than 86% CTC removal over 24 h. Compared with dispersed MOF-derived powder catalysts, N-101-NFF offers direct catalyst recovery and fixed-bed compatibility without requiring an additional magnetic or solid–liquid separation step. Nevertheless, the solvent- and energy-intensive preparation and the currently limited duration of continuous-flow evaluation remain important considerations for future scale-up. This work provides an effective strategy for designing recoverable MOF-derived monolithic catalysts for PMS-based treatment of antibiotic-contaminated wastewater.

## Figures and Tables

**Table 1:** Comparison of representative PMS-based systems for tetracycline-class antibiotic degradation.

Catalyst/System	Catalyst Configuration	Key Operating Conditions	Degradation Performance	Reusability/Continuous-Flow Performance	Ref.
N-101-NFF/PMS	Fe/Ni monolith	CTC = 50 mg/L; PMS = 0.08 mmol/L; catalyst area = 1 cm^2^; pH = 4.86; 60 min	90.3%	88.6% after five cycles; >86% over 24 h continuous flow	This work
N-101-NFF(Co_0.05_)/PMS	Fe/Ni/Co monolith	CTC = 50 mg/L; PMS = 0.08 mmol/L; catalyst area = 1 cm^2^; 60 min	95.7%	90.5% after 5 cycles; >90.5% during 24 h continuous flow	[52]
Fe-NPC-600/PMS	*N*-doped Fe-MOF-derived magnetic powder catalyst	TC; Fe-NPC-600 = 15 mg; PMS = 30 mg; 25 °C; 20 min	Approximately 90%	>80% after four cycles; continuous flow not reported	[8]
Co@N-MC/PMS	Co/*N*-doped magnetic porous carbon powder	CTC = 60 mg/L; catalyst = 1 g/L; PMS = 1 g/L; pH 11; UV 254 nm; 60 min	91.27%	Stable performance; Co leaching of 0.308 mg/L; no continuous-flow data	[53]

Because the operating conditions varied substantially among the reported studies, the degradation efficiencies should not be interpreted as a direct ranking of catalyst performance.

## Data Availability

The data presented in this study are available upon request from the corresponding author.

## Supplementary Materials

The following supporting information can be downloaded at: https://www.mdpi.com/article/10.3390/molecules31162884/s1, Text S1. Characterization technique; Figure S1. Effects of water matrices (a), humic acid (HA) concentration (b), and catalyst reusability (c) on CTC degradation in the N-101-NFF/PMS system. Operating conditions: initial CTC concentration = 50 mg/L, PMS concentration = 0.08 mmol/L, initial pH = 4.86, and effective catalyst area = 1 cm^2^; Figure S2. Electrochemical impedance spectroscopy (EIS) Nyquist plots (a), cyclic voltammograms (CV) (b), linear sweep voltammetry (LSV) curves (c), and chronoamperometric current-time (i-t) curves (d) of 101-NFF and N-101-NFF in 0.5 mol/L Na_2_SO_4_ electrolyte. The scan rate used for the CV measurements was 50 mV/s; Table S1. Physicochemical properties of different water matrices; Table S2. Composition of N-101-NFF surface elements and groups before and after the reaction; Table S3. Ecotoxicity of CTC and its intermediates.
