# Supplementary material for: Investigation of the Performance and Mechanism of an N-Doped Monolithic Fe/Ni-Based Catalyst for PMS Activation Toward Chlortetracycline Degradation in Water
Source: Molecules. 2026 Aug 18;31(16):2884. doi: 10.3390/molecules31162884 (PMC13515415; doi:10.3390/molecules31162884)
Supplement: Supplementary file 1 [file molecules-31-02884-s001.zip › molecules-4429410-supplementary.pdf]

Supporting Information

Investigation on the performance and mechanism of an N-doped monolithic Fe/Ni-based catalyst for PMS activation toward chlortetracycline degradation in water

Yiqiong Yang, Juan Han, Cui Wang, Pingchuan Yang, Panchen Li, Xiaodong Zhang*

School of Environment and Architecture, University of Shanghai for Science and Technology, Shanghai 200093, China

* To whom correspondence should be addressed. Tel. +86 15921267160, Fax. +86 021 55275979

E-mail address: fatzhxd@126.com

Text S1. Characterization technique

X-ray diffraction (XRD):

X-ray diffraction (XRD) patterns were recorded on a D8 ADVANCE diffractometer (Bruker, Karlsruhe, Germany) using Cu Kα radiation (λ = 0.15418 nm) at 40 kV and 40 mA. The diffraction patterns were collected over a 2θ range of 10-80° at a scanning rate of 0.8° min^{-1}.

Scanning electron microscopy coupled with energy-dispersive X-ray spectroscopy (SEM-EDS):

The distribution of elements in the material is observed by energy spectrometer combined with scanning electron microscopy (Jeol JSM-7800F, Japan) at the acceleration voltage of 18-20 kV, and the surface morphology of the catalyst is analyzed.

Transmission electron Microscopy (TEM) and high-resolution Transmission electron microscopy (HRTEM):

Transmission electron microscopy (TEM) and high-resolution TEM (HRTEM) were performed using a Tecnai G2 F20 microscope (FEI Company, Hillsboro, OR, USA) to observe the morphology, crystal structure, and lattice fringes of the monolithic catalyst.

X-ray photoelectron Spectroscopy (XPS):

X-ray photoelectron spectroscopy (XPS, Axis Ultra DLD X, Kratos Analytical Ltd., Manchester, UK) with Al Kα radiation (hν = 1486.6 eV) was performed to acquire XPS survey and high-resolution spectra of the monolithic catalyst, aiming to analyze its surface elemental composition and chemical states.

Fourier Transform Infrared Spectroscopy (FT-IR):

A small amount of sample was scraped from the monolithic catalyst, mixed with spectroscopic-grade KBr, ground thoroughly, and pressed into pellets. FT-IR spectra were recorded using a Nicolet iS50 spectrometer (Thermo Fisher Scientific, Waltham, MA, USA) over the range of 4000–400 cm^{-1}.

Electrochemical measurements were conducted using a CHI660C electrochemical workstation (CH Instruments, Shanghai, China).

EPR spectra were recorded using an EMX Plus spectrometer (Bruker, Karlsruhe, Germany).

CTC degradation intermediates were analyzed using an LC–MS system consisting of a Waters 2695 liquid chromatograph coupled with a Waters ZQ2000 mass spectrometer (Waters Corporation, Milford, MA, USA).

Table S1. Physicochemical properties of different water matrices

Water matrix	pH	Electrical conductivity (mS cm^{-1})	TOC (mg/L)	Turbidity (NTU)
Deionized water	6.45	0.00	0.07	0.00
Tap water	6.32	0.24	0.76	0.20
Natural water	6.28	0.33	6.17	17.4

Table S2. Composition of N-101-NFF surface elements and groups before and after the reaction

Elements	Combined state	Fresh（%）	After reaction（%）
Fe 2p	Fe^0	8.56	2.37
	Fe^{2+}	56.33	50.15
	Fe^{3+}	35.11	47.48
Ni 2p	Ni^0	15.94	7.16
	Ni^{2+}	53.47	62.93
	Ni^{3+}	30.59	29.91
C 1s	C=C/C-C	48.13	39.64
	C=N	38.63	42.49
	C-O/C-N	13.24	17.87
N 1s	pyridinic N	34.74	33.56
	Fe-N	31.61	32.47
	pyrrolic N	33.65	33.97
O 1s	C=O	21.36	4.82
	C-O	23.71	22.49

Table S3. Ecotoxicity of CTC and its intermediates

Number	m/z	Structure	predicted acute toxicity (LC_{50}, mg/L)	Bioaccumulation factor	Developmental toxicity	Mutagenic
CTC	515	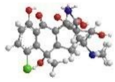	3.63	0.33	0.87	0.65
A1	444	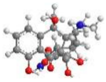	10.05	0.16	0.86	0.61
A2	399	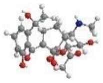	11.51	0.53	0.82	0.67
A3	294	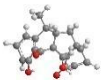	0.16	6.52	0.94	0.18
A4	280	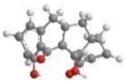	0.13	9.14	1.02	0.59
A5	264	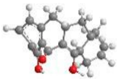	0.11	23.18	0.95	0.79
B1	449	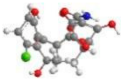	0.15	1.07	0.88	0.63
B2	465	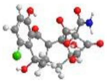	2.77	0.21	0.86	0.65
B3	463	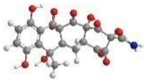	-	0.04	0.8	0.69
B4	413	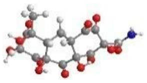	-	0.34	0.69	0.63
C1	112	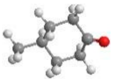	199.81	8.45	0.24	0.09
C2	130	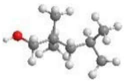	111.23	11.14	0.48	0.44
C3	150	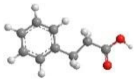	50.43	2.56	0.69	-
C4	88	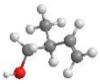	79.26	3.92	0.75	0.29
C5	114	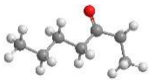	100.96	12.38	0.63	-
C6	92	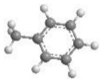	31.08	66.55	0.28	-

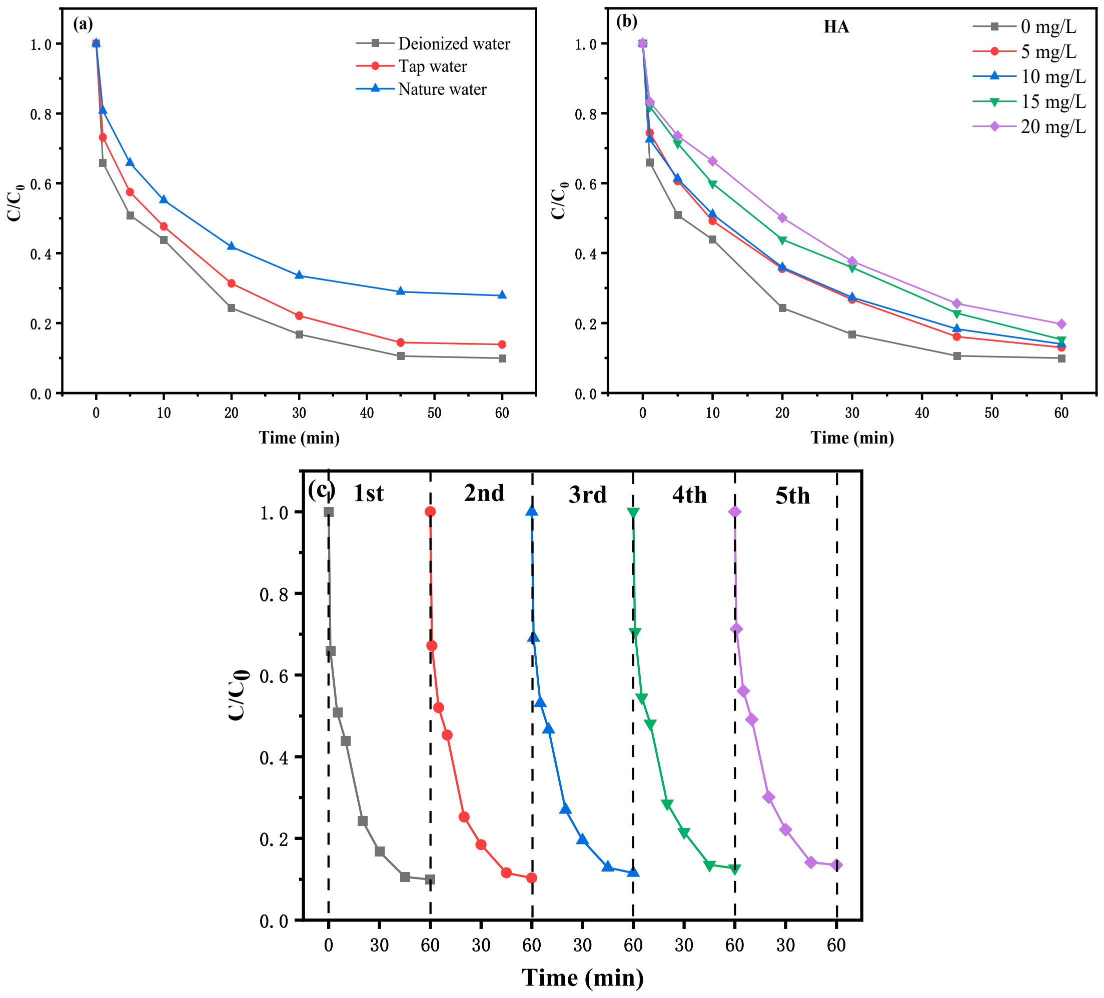

Figure. S1. Effects of water matrices (a), humic acid (HA) concentration (b), and catalyst reusability (c) on CTC degradation in the N-101-NFF/PMS system. Operating conditions: initial CTC concentration = 50 mg/L, PMS concentration = 0.08 mmol/L, initial pH = 4.86, and effective catalyst area = 1 cm^2.

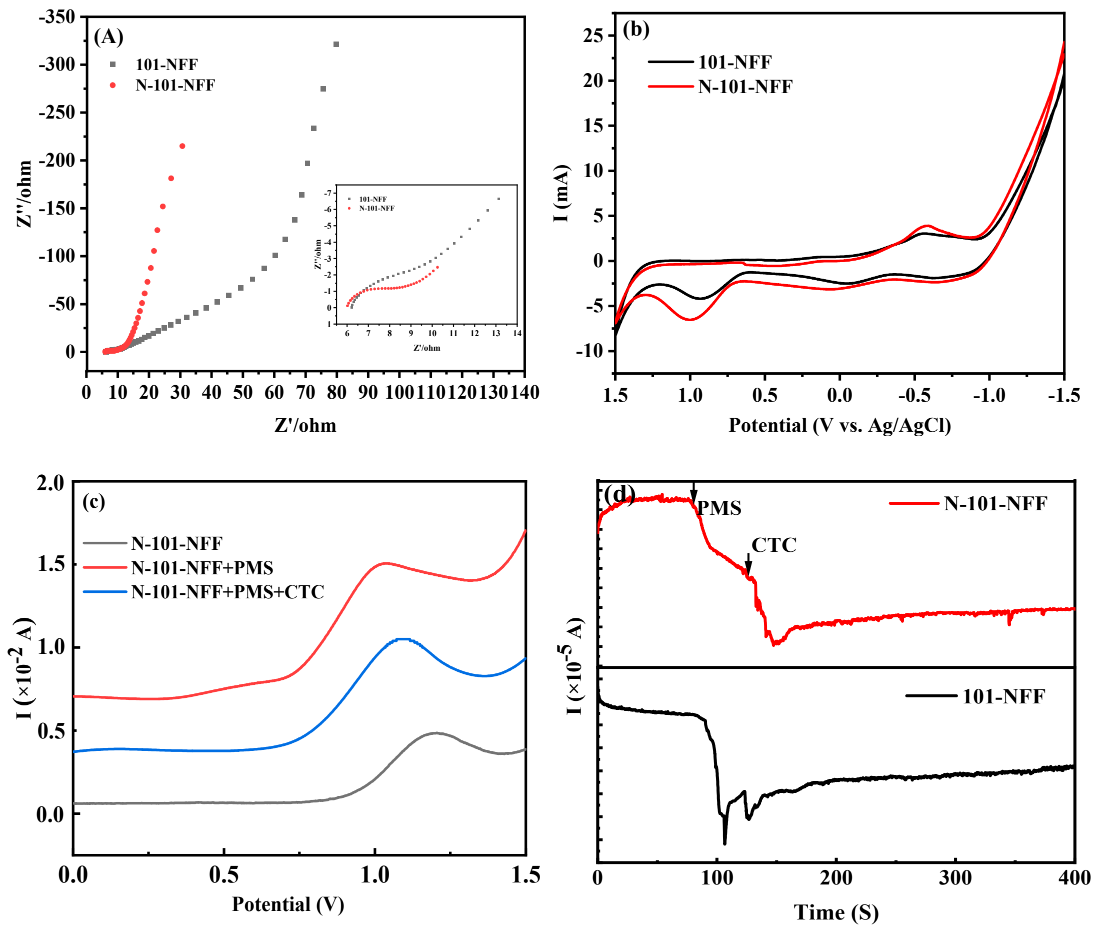

Figure. S2. Electrochemical impedance spectroscopy (EIS) Nyquist plots (a), cyclic voltammograms (CV) (b), linear sweep voltammetry (LSV) curves (c), and chronoamperometric current-time (i-t) curves (d) of 101-NFF and N-101-NFF in 0.5 mol/L Na_2SO_4 electrolyte. The scan rate used for the CV measurements was 50 mV/s.
